# Resilient Seas: Environmental Drivers of Larval Fish Assemblages in the Gulf of Tortugas, Colombian Pacific

**DOI:** 10.3390/life16091468

**Published:** 2026-09-02

**Authors:** Diego Fernando Córdoba Rojas, Juan José Gallego Zerrato, Alan Giraldo

**Affiliations:** 1 Programa Académico de Doctorado en Ciencias del Mar, Departamento de Biología, Facultad de Ciencias Naturales y Exactas, Universidad del Valle, Cali 760032, Colombia; diego.cordoba.rojas@correounivalle.edu.co; 2 Grupo de Investigación en Ciencias Oceanográficas, Departamento de Biología, Facultad de Ciencias Naturales y Exactas, Universidad del Valle, Cali 760032, Colombia; juan.j.gallego@correounivalle.edu.co; 3 Programa Académico de Doctorado en Ciencias Biología, Departamento de Biología, Facultad de Ciencias Naturales y Exactas, Universidad del Valle, Cali 760032, Colombia

**Keywords:** coastal connectivity, Eastern Tropical Pacific, ecosystem resilience, hydrographic variability, ichthyoplankton, ENSO, spatiotemporal dynamics

## Abstract

Nursery habitats sustain fish populations by supporting early life stages and buffering environmental variability, yet their functioning under climate oscillations remains poorly understood in the Eastern Tropical Pacific. We analyzed ichthyoplankton assemblages in the Gulf of Tortugas, Colombia (2010–2020), to assess taxonomic composition, spatiotemporal variability, and environmental drivers. ENSO cycles and local oceanographic conditions were evaluated as determinants of nursery function and resilience using richness estimators, diversity indices, Caswell’s neutral model, and multivariate approaches (PERMANOVA, PERMDISP, CAP, BIOENV). Assemblages were dominated by *Cetengraulis mysticetus*, with recurrent contributions from Sciaenidae, Haemulidae, Gerreidae, and Carangidae—families of artisanal fisheries importance. ENSO phases explained interannual variability: El Niño years reduced diversity and weakened retention, whereas La Niña years supported more balanced assemblages. Spatial heterogeneity revealed central retention zones and peripheral diversity hotspots. Environmental gradients, particularly chlorophyll-a, zooplankton biomass, transparency, and pH, consistently structured larval distribution, reflecting the coupling of productivity, water quality, and circulation. These findings demonstrate that the Gulf of Tortugas functions as a dual nursery system and ecological node of resilience, underscoring the importance of protecting such habitats for ecosystem-based fisheries management and biodiversity conservation in a changing ocean.

## 1. Introduction

The early developmental stages of fishes—eggs and larvae (ichthyoplankton)—are a critical component of marine biodiversity. They contribute biomass, mediate trophic interactions, and sustain ecosystem functioning [1,2,3,4,5]. Ichthyoplankton studies provide essential insights into adult fish biology and ecology [6,7,8,9,10,11]. They also inform fisheries science by identifying spawning areas, estimating adult population sizes, and predicting recruitment success [12,13,14,15,16]. In the context of global change, ichthyoplankton research has become increasingly relevant for understanding how biodiversity and ecosystem functioning respond to climatic variability and anthropogenic pressures.

Coastal larval fish assemblages differ markedly from oceanic ones due to complex physical, chemical, and biological interactions. Key drivers include temperature, salinity, currents, food availability, and reproductive strategies of adult populations [11,17,18,19,20]. Oceanographic features such as eddies, stratification, and freshwater inputs further modulate larval survival and distribution [21,22,23,24,25,26]. At broader scales, ichthyoplankton assemblages respond to climatic oscillations, particularly El Niño and La Niña events, which alter productivity, circulation, and recruitment dynamics [27,28,29,30,31,32,33,34]. While some studies emphasize ENSO as a dominant driver of larval variability, others highlight the buffering role of local geomorphology and reproductive strategies [35,36], underscoring ongoing debate about the relative importance of global versus local processes.

Nursery habitats are critical ecological nodes that sustain early life stages of marine fishes, providing refuge, food resources, and favorable hydrographic conditions that enhance survival and recruitment [37,38,39]. Estuarine transition zones, coastal gulfs, and mangrove systems are particularly important because they integrate oceanic and continental influences, thereby supporting both shelf-associated and pelagic taxa. These habitats function not only as biodiversity reservoirs but also as buffers against environmental variability, maintaining ecosystem resilience and contributing to fisheries productivity [40]. In the nearshore area of the Eastern Tropical Pacific (ETP), larval assemblages are frequently dominated by Engraulidae, Sciaenidae, Haemulidae, and Carangidae, reflecting the influence of ENSO variability, upwelling regimes, and estuarine–ocean interactions [36,41,42,43,44,45,46,47]. These findings underscore the ETP as a dynamic biogeographic unit where coastal gulfs and transition zones function as nurseries, linking local assemblages to regional resilience.

Globally, nursery habitats play a critical ecological role. In Bahía Magdalena, Mexico, ichthyoplankton assemblages respond to ENSO variability while maintaining connectivity with regional fisheries [28,29,48,49,50]. Chilean fjords sustain early developmental stages through circulation and retention processes [31,51]. Comparable mechanisms occur in South African estuaries [24,52,53], Brazilian coastal systems [26,54,55], Mediterranean stratified waters [19,20,56], and Indian Ocean and Taiwan Bank upwelling regions [23,57,58]. These parallels highlight the global importance of nursery habitats as ecological keystones and socio-economic assets.

In Colombia, ichthyoplankton research dates back to the 1980s [59,60,61,62,63,64,65,66]. Recent studies emphasize ENSO variability, intraannual dynamics, and estuarine processes [32,36,47,67,68,69]. However, long-term continuity in Colombian ichthyoplankton studies has been limited, largely due to short temporal coverage (typically 1–3 years), restricted spatial scope to single estuaries or bays, irregular sampling frequency, and methodological inconsistencies across campaigns. These constraints have hindered robust assessments of spatiotemporal variability and its implications for biodiversity and fisheries resilience.

Within this context, the ecological role of coastal gulfs in the Colombian Pacific is still poorly understood. The Gulf of Tortugas, located in the Eastern Tropical Pacific, represents a coastal system influenced by oceanic inflows, riverine discharges, and tidal dynamics. Major rivers such as the San Juan, Raposo, Mayorquín, and Cajambre contribute substantial freshwater inputs, with peak discharges during the wet season (April–November), while tidal dynamics are semidiurnal with an average range of 3–4 m. This hydrographic complexity suggests a high potential for nursery function, as the interaction of riverine inflows, tidal mixing, and oceanic circulation creates favorable conditions for larval retention and dispersal. Its hydrographic complexity suggests a high potential for nursery function, yet long-term assessments of ichthyoplankton assemblages in this region are scarce. Understanding how local biophysical drivers interact with large-scale climatic oscillations is therefore essential for evaluating nursery capacity and resilience under climate variability. The present study addresses this gap by analyzing ichthyoplankton assemblages in the Gulf of Tortugas through a decade of monitoring, thereby providing one of the most comprehensive assessments of nursery function in the Colombian Pacific. Our approach integrates spatial heterogeneity, local biophysical modulation, and large-scale climatic forcing to evaluate how nursery capacity and resilience are sustained under fluctuating environmental conditions.

Specifically, we test three contrasting hypotheses: (1) The Gulf of Tortugas functions as a dual nursery, with central stations—defined a priori as sites in the innermost portion of the Gulf, equidistant from coastal margins and riverine inflows where retention cells were expected—acting as retention zones dominated by few species, while peripheral stations—located near the outer margins and estuarine/oceanic transition zones where dispersive pathways and stronger hydrographic gradients were observed—serve as diversity hotspots. (2) Local biophysical drivers (transparency, chlorophyll a, zooplankton biomass, pH) modulate larval distribution and assemblage structure. (3) ENSO variability, expressed through Oceanic Niño Index (ONI) phases, drives interannual shifts in diversity, richness, and taxonomic composition, with El Niño suppressing diversity and La Niña enhancing resilience.

By contrasting these hypotheses, this study contributes to understanding marine biodiversity and ecological functioning in a changing ocean, offering empirical evidence of how nursery habitats sustain ecosystem processes, support biodiversity conservation, and reinforce fisheries sustainability under climate variability.

## 2. Materials and Methods

### 2.1. Study Area

**Figure 1:**
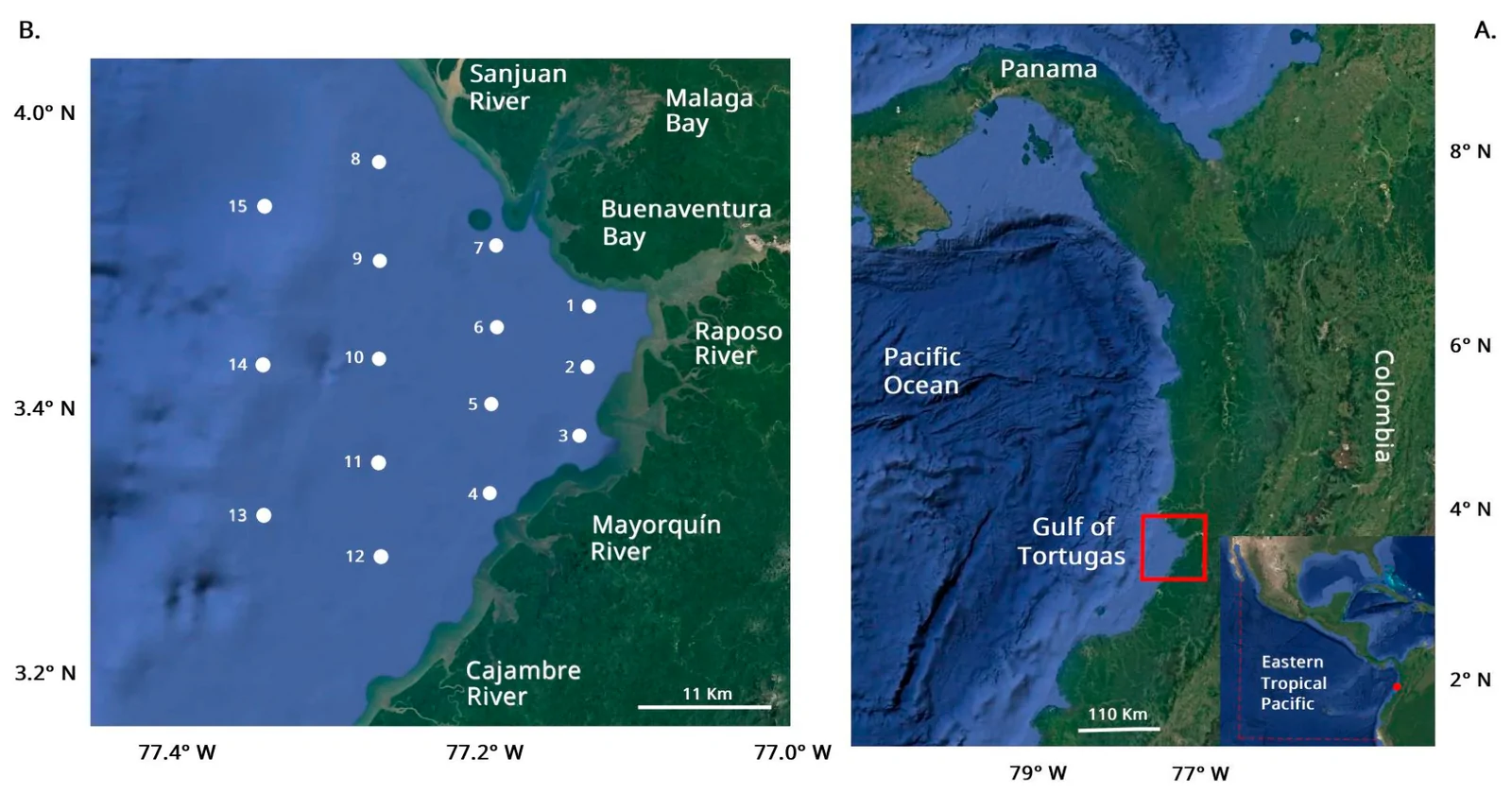
(**A**). Location of the Gulf of Tortugas in the Eastern Tropical Pacific (red dot) and along the Colombian Pacific coast (red square). (**B**) Detailed coastal view of the Gulf of Tortugas showing sampling stations (1–15). Image source: Google Earth^®^, Landsat/Copernicus, (accessed 3 March 2026), modified by the authors. Image reproduced in accordance with Google Earth Guidelines for fair academic and research use.

Ichthyoplankton sampling was conducted in the Gulf of Tortugas, located in the Colombian Eastern Tropical Pacific (3°20′–4°00′ N; 77°00′–77°40′ W), between 2010 and 2020 by the Oceanographic Sciences Research Group at Universidad del Valle. The Gulf is influenced by oceanic inflows, riverine discharges, and tidal dynamics. Seasonal thermocline shifts and freshwater inputs create favorable conditions for larval retention and dispersal, highlighting its potential as a nursery habitat (Figure 1).

The region sustains early life stages of both pelagic and demersal fishes, many of which are of artisanal fisheries importance [70]. Oceanographic variability is strongly influenced by seasonal upwelling from the Panama Bight and by ENSO cycles. El Niño phases are typically associated with thermocline deepening and reduced productivity, whereas La Niña phases correspond to enhanced upwelling and increased biodiversity. To capture this variability, a standardized sampling grid was established. From 2010 to 2014, the grid comprised 12 equidistant stations. In 2015, it was expanded to 15 stations, encompassing coastal, estuarine, and oceanic transition zones (Figure 1).

### 2.2. Sampling Design

Zooplankton collections were performed through oblique bongo net tows at all stations. Nets had a mouth diameter of 30 cm and mesh sizes of 300 µm and 500 µm. Samples from both meshes were pooled for analysis. Tow durations and depths were standardized across campaigns. Filtered water volume was estimated using a General Oceanics^®^ flowmeter (2010–2012) and a Hydrobios^®^ flowmeter (2014–2020); no sampling was conducted in 2013 (Table S1).

Samples were preserved in buffered formalin (10% final concentration, prepared with borax and seawater). Fish larvae were sorted and identified to the lowest possible taxonomic level using the ichthyoplankton guide for the Colombian Pacific [71] and the atlas of Moser [72]. Abundances were standardized to filtered water volume and expressed as density (individuals per cubic meter, ind/m^3^).

Physicochemical parameters (temperature, salinity, dissolved oxygen, and pH) were measured at 0, 10, 30, and 50 m using a YSI Pro Plus multiparameter (2010–2014) and a Hanna HI9829 multiparameter (2015–2020). Continuous temperature and salinity data from surface to 80 m or near bottom were obtained using a Seabird 19 profiler (2010–2014) and a CTD YSI Castaway profiler (2015–2020). Instrument replacements (flowmeters, multiparameter probes, CTD systems) were calibrated against reference standards and cross-checked with overlapping measurements during transition campaigns to ensure comparability of long-term environmental data and minimize bias. Transparency was assessed with a Secchi disk. Chlorophyll *a* concentration was determined spectrophotometrically following the Lorenzen method [73], after pigment extraction in 90% acetone and absorbance readings at the recommended wavelengths with correction for turbidity. Total mesozooplankton biomass, representing food availability, was quantified from pooled subsamples dried at 60 °C for 24 h and weighed in triplicate with an analytical balance (precision 0.0001 g) [74].

Surface circulation (1 m) was assessed using drifting buoys equipped with GPS receivers, deployed for 30 min at each station. Buoy positions were recorded at deployment and recovery, yielding a displacement vector from which drift velocity and direction were calculated using the Haversine formula, which provides accurate great-circle measurements on a spherical surface. Although Vincenty’s formula offers higher precision by accounting for Earth’s ellipsoidal geometry, its computational complexity was unnecessary for short-range drift analyses. Drift vectors were subsequently integrated into a GIS to delineate zones of inflow, retention, and dispersal.

Vertical stability was quantified using the Brunt–Väisälä frequency ($\beta =N^{2}=-g∆\rho /\rho ∆Z$), which measures buoyancy frequency. Higher β values indicate stronger stratification and reduced vertical mixing, while lower values reflect increased turbulence and homogenization. Horizontal stratification was evaluated using the potential energy anomaly (Φ), following Lee et al. [22]. Values < 10 J m^−3^ indicate mixed waters, 10–20 J m^−3^ frontal waters, and ≥20 J m^−3^ stratified waters. Together, β and Φ provided a comprehensive characterization of vertical and horizontal stability, circulation, and mixing processes.

### 2.3. Data Analysis

Interannual comparisons were conducted at the locality level, with diversity and assemblage metrics standardized to account for variation in the number of stations sampled per campaign. Sampling representativeness was evaluated using a suite of non-parametric richness estimators (Chao1, Jackknife1, Bootstrap), which provided complementary assessments of expected species richness and ensured adequate coverage of assemblage diversity across campaigns. These estimators allowed us to determine whether observed richness reflected true taxonomic breadth or was constrained by sampling effort, thereby strengthening the reliability of long-term comparisons. Diversity was further quantified through classical univariate indices, including Shannon diversity (H′) and Pielou’s evenness (J′), each offering distinct perspectives on richness, dominance, and evenness within assemblages. Caswell’s neutral model statistic (V) was calculated to test deviations from neutral expectations [75]. Negative values indicated diversity below neutral expectations, consistent with reproductive aggregation, while positive values suggested more balanced assemblages.

Multivariate analyses were conducted in PRIMER v6 [76]. Dispersion was examined using PERMDISP to ensure homogeneity of variance [77]. Environmental datasets were normalized and square-root transformed, while ichthyoplankton densities were log-transformed [log10(x + 1)]. Taxa with an occurrence frequency greater than 5% across sampling stations were retained for multivariate analysis, reducing the influence of rare taxa and improving the interpretability of assemblage patterns. A Bray–Curtis similarity matrix was generated to quantify compositional dissimilarities.

PERMANOVA was applied to evaluate spatial and interannual variability in ichthyoplankton assemblages [78,79]. The analysis incorporated campaign as a random factor and station nested within zone as a fixed factor, using 9999 permutations constrained by the design to account for repeated sampling and spatial dependence. Zones were delineated a priori according to geographic position within the Gulf of Tortugas: inner stations located nearshore and influenced by riverine inputs, middle stations situated in the central portion of the gulf, and outer stations positioned toward the seaward margin adjacent to oceanic waters. Pairwise comparisons among significant factors were conducted using 9999 permutations to ensure robust statistical inference.

To explore relationships between assemblage structure and environmental drivers, BIOENV was employed to compare Bray–Curtis similarity matrices of species composition with normalized environmental datasets (temperature, salinity, chlorophyll *a*, and zooplankton biomass). Canonical Analysis of Principal Coordinates (CAP) was subsequently applied to identify the axes that best explained groupings and to visualize multivariate patterns. ENSO variability was incorporated into the analysis by including the Oceanic Niño Index (ONI) as a covariate, enabling direct evaluation of El Niño and La Niña influences on assemblage structure and diversity. Campaigns were classified into ENSO phases according to ONI thresholds. This integrative approach allowed us to assess both local environmental forcing and large-scale climatic oscillations as determinants of larval fish distribution. Collinearity among environmental variables was assessed using variance inflation factors and pairwise correlations, and highly correlated predictors were excluded or interpreted with caution.

## 3. Results

### 3.1. Taxonomic Composition and Richness

A total of 7379 fish larvae were collected, representing 133 morphospecies. Thirty-nine species (29%) were fully identified, while 278 individuals (4%) remained unclassified (Table S2). The assemblage included 100 genera, 34 families, and 20 orders, suggesting broad taxonomic coverage. Across all nine campaigns, *Cetengraulis mysticetus* (Engraulidae) accounted for nearly half of the total abundance (48%). Sciaenidae (18%), Gobiidae (13%), and Haemulidae (3.3%) also contributed, although dominance varied among campaigns. Families of artisanal fisheries interest, such as Carangidae, Lutjanidae, and Centropomidae, were present in lower proportions. At the genus level, *Cetengraulis* remained consistently dominant, while *Isopisthus*, *Cynoscion*, *Stellifer*, and *Eucinostomus* appeared recurrently, suggesting connectivity between estuarine and coastal habitats.

Most larvae (98%) were in the preflexion stage, pointing to dependence on local circulation and retention processes. Richness estimators indicated that observed richness was close to predicted values: Chao 1 (observed) = 133 spp., Jackknife1 = 166 spp. (80% of predicted), and Bootstrap = 149 spp. (89% of predicted). Diversity indices suggested strong interannual variability. Campaigns in 2012 and 2019 tended toward higher richness and evenness, whereas 2017 was characterized by extreme dominance and reduced diversity despite high larval density. Caswell’s neutral model produced values that departed from neutrality in several campaigns, patterns consistent with reproductive aggregation and retention tendencies (Table 1).

### 3.2. Surface Circulation Pattern

**Figure 2:**
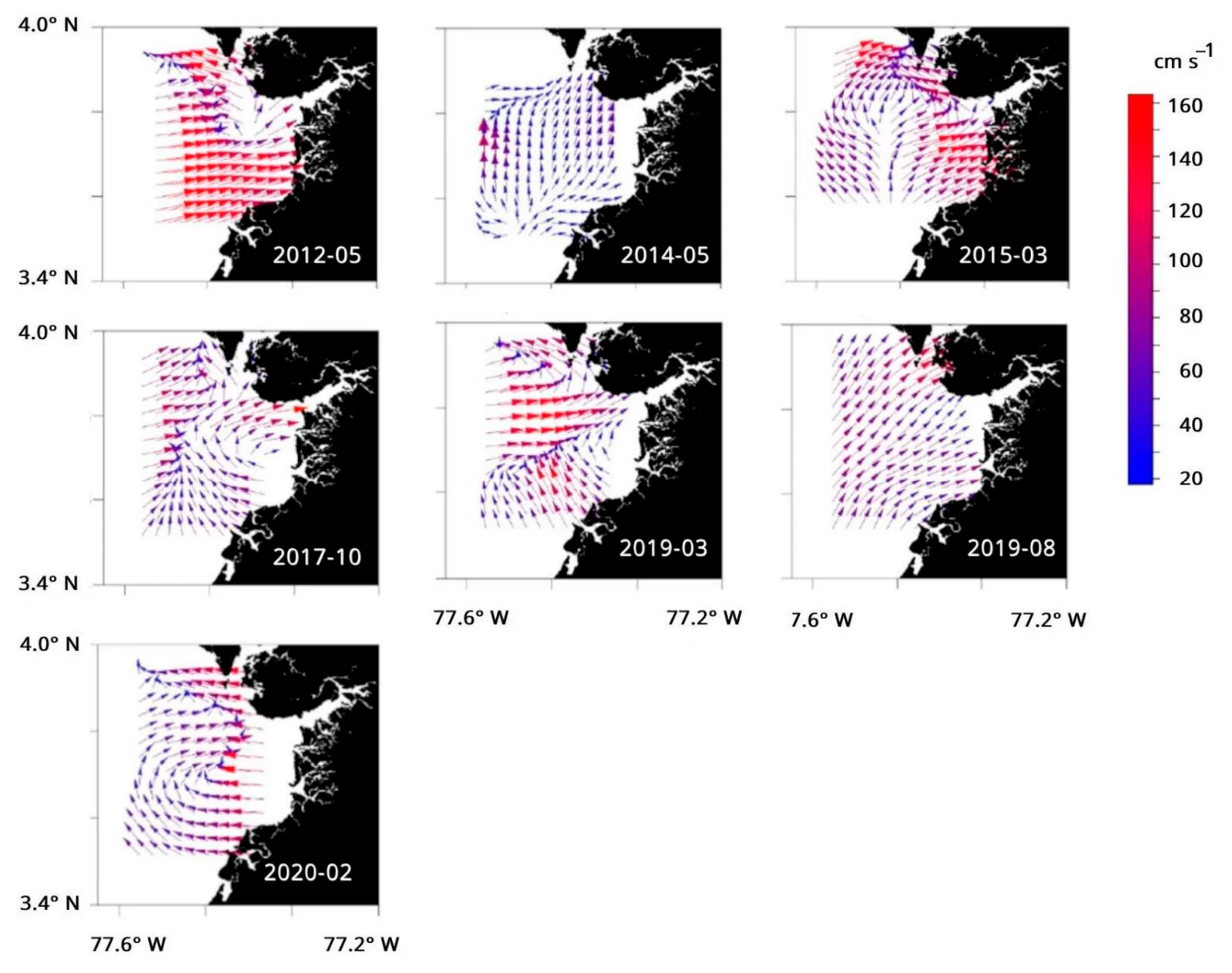
Surface circulation patterns in the Gulf of Tortugas for each sampling campaign, restricted to the years in which buoy-tracking data were available. Vector length indicates drift velocity, while arrow orientation denotes current direction.

Surface current dynamics showed spatial heterogeneity across the Gulf of Tortugas. Inflow zones were observed along the coastal margin, retention cells occupied the central region, and dispersive pathways extended toward oceanic transition areas (Figure 2). These features suggested a spatial mosaic of circulation regimes that may influence larval distribution.

GPS-tracked drifting buoys showed reduced displacement within retention cells, pointing to limited horizontal transport and a tendency toward larval aggregation. In contrast, dispersive pathways carried buoy trajectories toward oceanic stations, where assemblages appeared more taxonomically diverse and heterogeneous. Circulation features of this type were noted across campaigns. Retention cells seemed to function as potential biomass reservoirs, while dispersive pathways were associated with biodiversity through export and mixing. The coexistence of these regimes suggested that the Gulf may operate as a dual nursery system, combining localized retention with broader dispersal.

### 3.3. Local Biophysical Drivers and ENSO Variability

**Figure 3:**
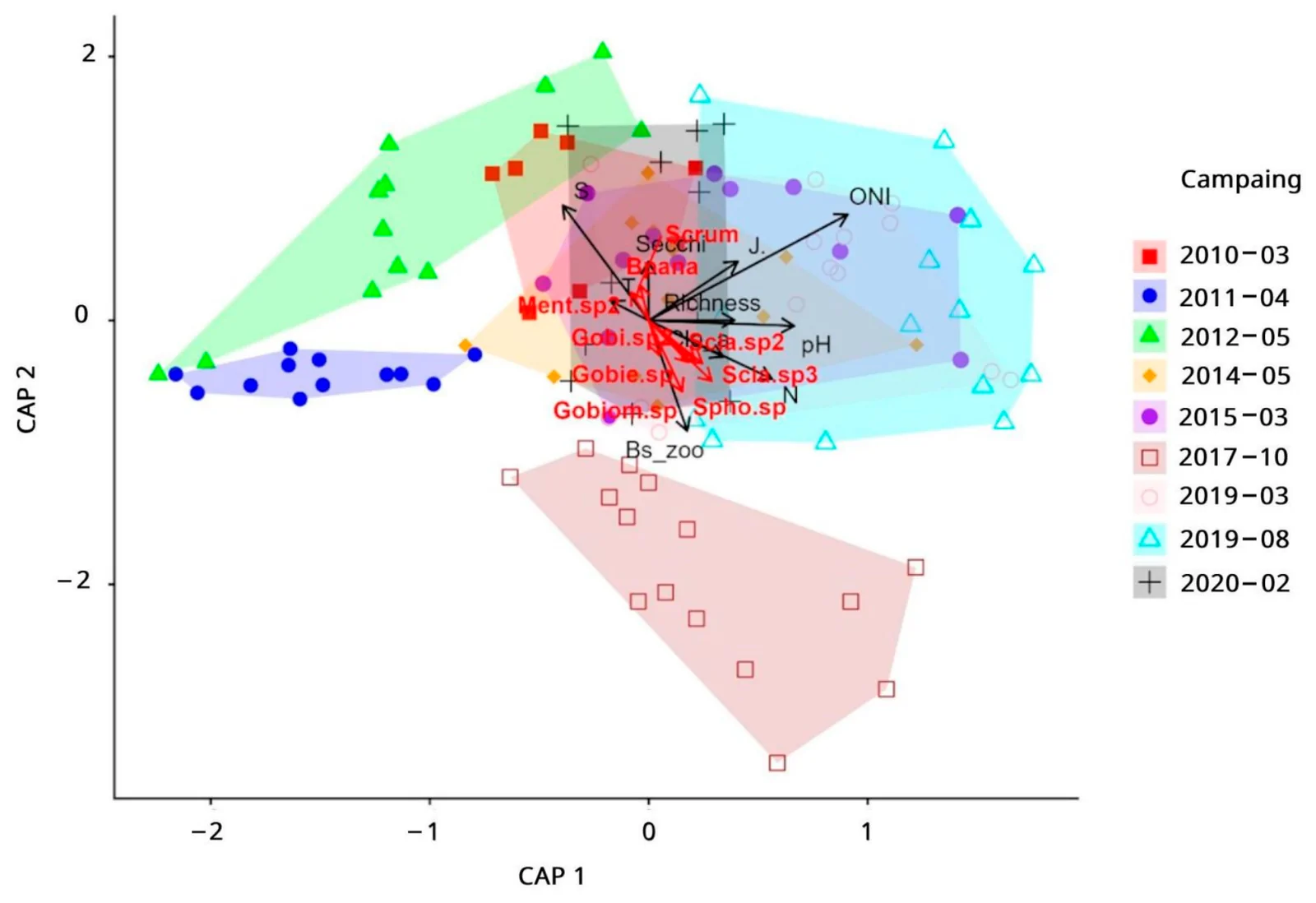
Canonical Analysis of Principal Coordinates (CAP) showing relationships between environmental variables and ichthyoplankton assemblages across sampling campaigns. Convex hulls delimit campaign groups, and vectors indicate environmental gradients and species associations, with ONI highlighting the influence of ENSO variability on assemblage structure.

Larval assemblages were closely linked to local biophysical conditions and modulated by ENSO variability. Stations with higher chlorophyll–a and zooplankton biomass supported elevated larval densities, while reduced transparency and lower pH were associated with assemblages dominated by few taxa. Chlorophyll-a, zooplankton biomass, transparency, and pH were consistently identified as important predictors across Spearman correlations, BIOENV, and CAP analyses, highlighting their central role in structuring larval assemblages. Spearman’s correlations (rs), calculated using station–campaign observations (*n* = 114) and based on raw data values (Tables S1 and S3), confirmed significant positive relationships between larval density and chlorophyll–a (rs = 0.62, *p* < 0.01) and zooplankton biomass (rs = 0.58, *p* < 0.01). Diversity indices declined under reduced transparency (rs = −0.47, *p* < 0.05) and lower pH (rs = −0.41, *p* < 0.05). BIO-ENV identified chlorophyll–a and transparency as the best predictors of assemblage composition (rs = 0.62), while CAP ordination emphasized gradients of productivity and water clarity as primary structuring axes (Figure 3).

**Figure 4:**
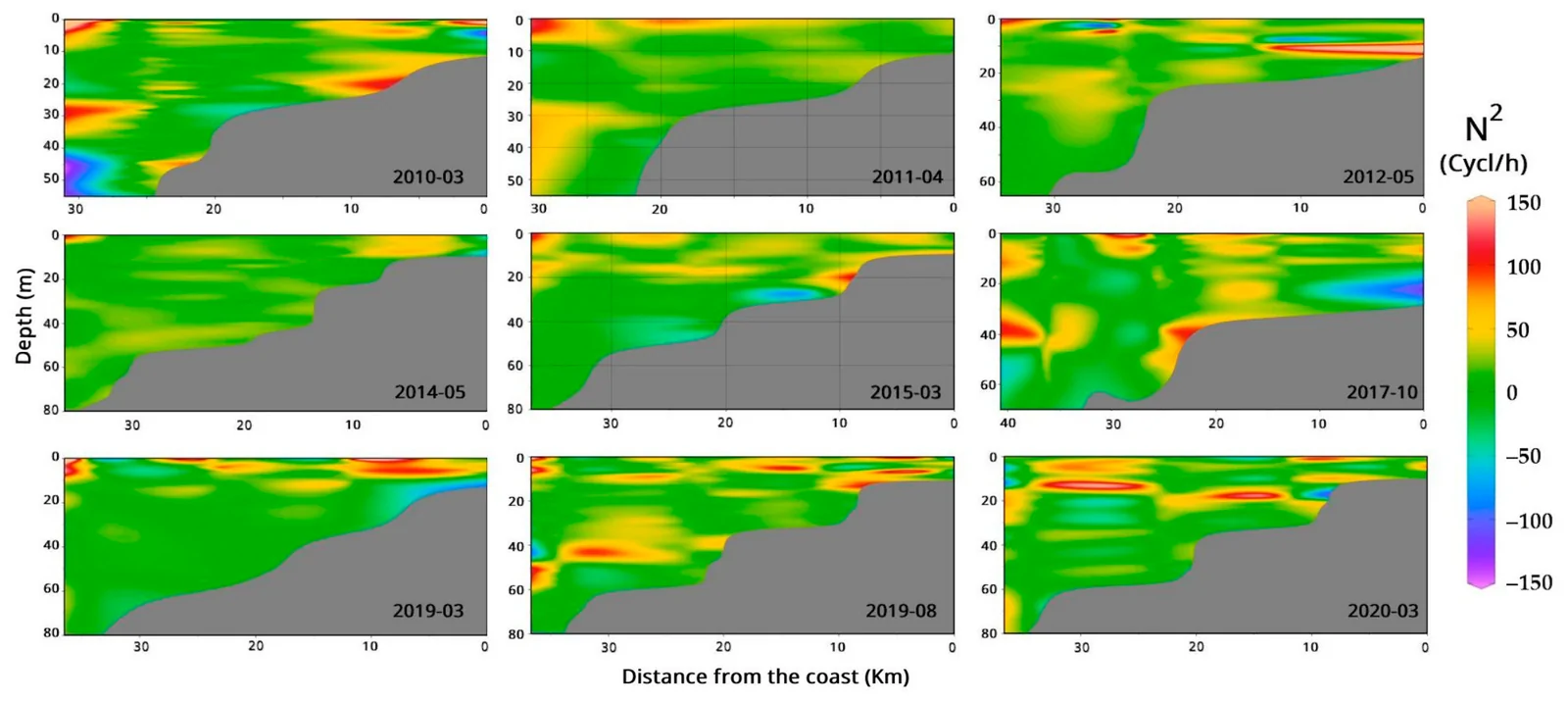
Vertical stratification in the Gulf of Tortugas expressed through Brunt–Väisälä frequency (N^2^). Profiles illustrate seasonal and interannual variability in water column stability between 2010 and 2020.

Seasonal variation in the water column further modulated these dynamics. Vertical stratification, expressed through the Brunt–Väisälä frequency (N^2^), revealed stronger stability during warm phases, with surface waters exceeding 28–30 °C and subsurface layers remaining cooler (<20 °C). During El Niño years (2015–2016), N^2^ values increased in the upper 20–40 m, indicating reduced vertical mixing and deeper thermoclines. These conditions coincided with diminished nutrient flux, weakened retention, and lower larval biomass. In contrast, La Niña years (2010–2011) exhibited lower N^2^ values, reflecting weaker stratification and enhanced vertical mixing. Shallower thermoclines promoted higher productivity and supported more diverse larval assemblages (Figure 4).

**Figure 5:**
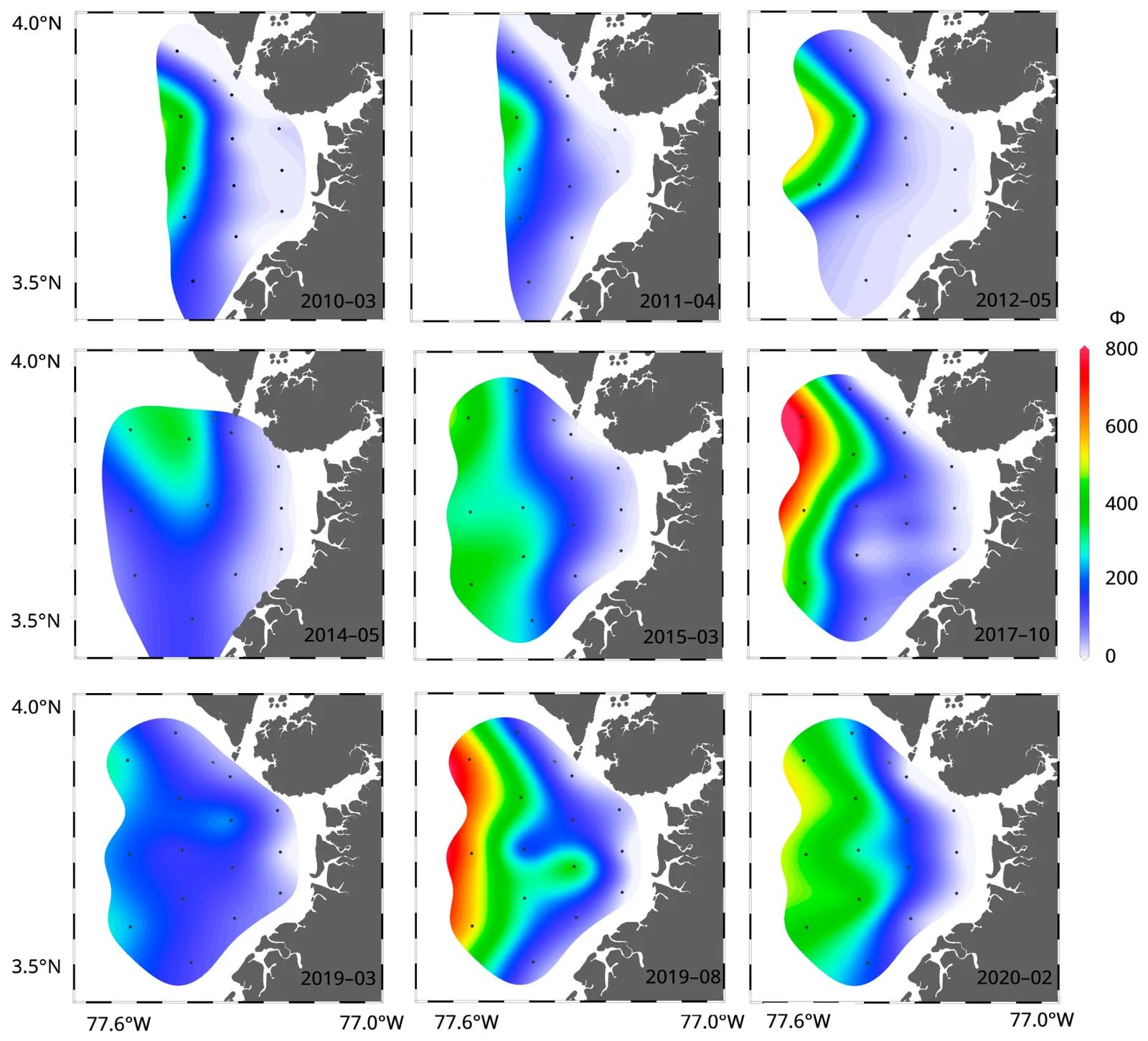
Horizontal stratification in the Gulf of Tortugas expressed through potential energy anomaly (Φ). Spatial distributions illustrate seasonal and interannual variability in hydrographic stability between 2010 and 2020.

Horizontal stratification also varied seasonally and interannually. El Niño campaigns (2015, 2019) were characterized by weakened inflow and reduced retention efficiency, with hydrographic gradients flattening across central stations. This homogenization limited dispersal pathways and concentrated biomass into retention zones. La Niña campaigns (2011, 2017) displayed stronger inflow and sharper horizontal gradients in temperature, salinity, and chlorophyll a. These gradients delineated retention cells in central areas and dispersive pathways toward peripheral stations, facilitating both biomass concentration and diversity export (Figure 5).

**Figure 6:**
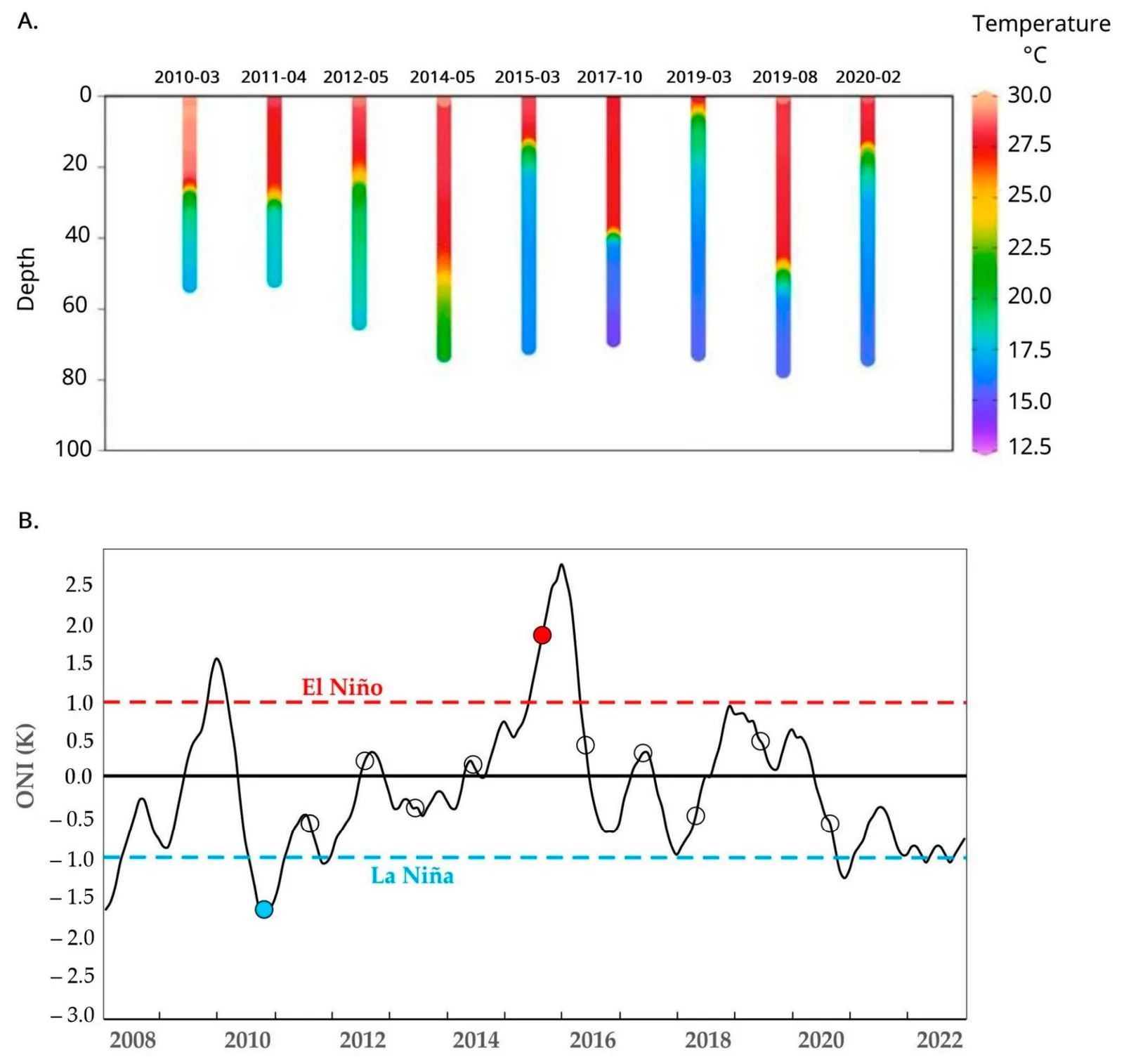
(**A**). Vertical temperature profiles (°C, averaged per campaign) observed during sampling in the Gulf of Tortugas between 2010 and 2020. Profiles illustrate seasonal and interannual variability in the thermal structure of the water column, with shoaling of the thermocline during March campaigns (2010, 2015, 2019) favoring higher diversity and balanced assemblages, and deepening during October 2017 and August 2019 associated with strong dominance. (**B**). Oceanic Niño Index (ONI) from 2008 to 2022, showing the timing and intensity of El Niño (red threshold, +1.5 K) and La Niña (blue threshold, −1.5 K) events. Notable peaks include the strong El Niño of 2015 and the La Niña of 2010. Together, these panels contextualize the influence of ENSO-related climatic oscillations and seasonal hydrography on larval assemblage variability and nursery function in the Gulf of Tortugas.

Vertical temperature profiles revealed marked stratification during warm phases (Figure 6A). Surface waters reached 28–30 °C, while subsurface layers remained cooler (<20 °C). During El Niño years (2015–2016), thermocline deepening reduced nutrient availability, coinciding with weakened retention and lower larval biomass. In contrast, La Niña years (2010–2011) were characterized by shallower thermoclines and enhanced vertical mixing. These conditions promoted higher productivity and supported more diverse larval assemblages (Figure 6A).

In a broader climatic context, ONI oscillations delineated warm (El Niño) and cool (La Niña) phases that influenced nursery function. The strong El Niño of 2015 coincided with suppressed diversity and marked dominance by *Cetengraulis mysticetus* (>60% of total abundance). These conditions were accompanied by weakened retention, reduced chlorophyll–a, and diminished zooplankton biomass (Table 1 and Table S3). In contrast, La Niña phases facilitated recovery, with richness increasing by 25–30% and Shannon diversity values nearly doubling compared to El Niño years (Table 1 and Table S3).

ENSO phases (Figure 6B) also affected circulation intensity and ecological outcomes. During El Niño campaigns (2015), inflow weakened and retention declined (Figure 2), coinciding with lower larval biomass (Table S3). These years were marked by deeper thermoclines and reduced productivity, factors that likely contributed to diminished survival in central nursery zones. La Niña campaigns (2011) showed stronger inflow and enhanced retention (Figure 2). These conditions supported improved survival and greater diversity in peripheral stations, associated with shallower thermoclines, enhanced mixing, and increased resource availability. The alternation between El Niño suppression and La Niña recovery suggested that nursery function in the Gulf of Tortugas is closely linked to climatic oscillations, with resilience emerging during cooler phases.

### 3.4. Dual Nursery Function and Integrative Patterns

**Figure 7:**
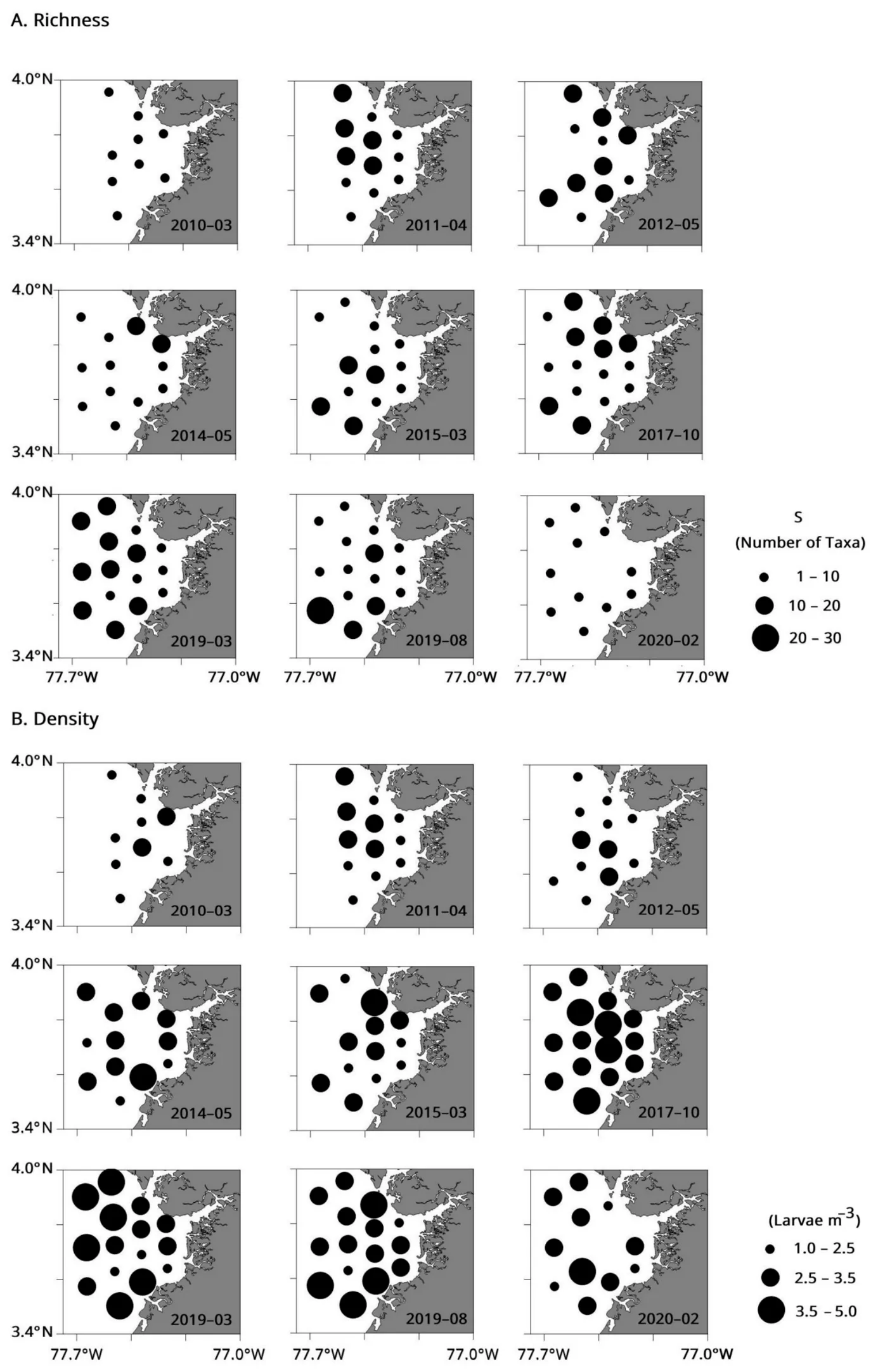
(**A**). Spatial distribution of species richness (S) across sampling campaigns in the Gulf of Tortugas, expressed as the number of morphospecies per station. (**B**). Spatial distribution of larval density (N, individuals per cubic meter), standardized to filtered water volume. Circle size in each panel reflects relative values of richness or density, allowing comparison of temporal and spatial variability in ichthyoplankton assemblages.

Ichthyoplankton assemblages showed spatial partitioning consistent with a dual nursery function. Central stations within retention cells were characterized by reproductive aggregation, sustaining elevated larval densities dominated by *Cetengraulis mysticetus*. Richness and evenness at these stations were comparatively low. Peripheral stations, influenced by dispersive pathways, supported a broader array of families, including Carangidae, Gobiidae, Sciaenidae, and Engraulidae. These areas tended to function as diversity hotspots, contributing to ecological heterogeneity. Such heterogeneity may enhance resilience by maintaining taxonomic breadth across the system (Table 2, Figure 7).

PERMDISP detected differences in dispersion among campaigns (F = 4.78, df1 = 8, df2 = 105, *p(perm)* = 0.001). PERMANOVA indicated interannual variation in assemblage composition, with variability evident across campaigns, stations, and zones (*p* < 0.01) (Table 2). Diversity indices reflected these contrasts. Shannon diversity (H′) and Hill numbers (N1, N2) tended to be higher at peripheral stations. In contrast, Margalef richness (d) and Pielou’s evenness (J′) emphasized the dominance of few species in central zones (Table S3).

### 3.5. Integrative Results Summary

Long-term monitoring of fish larvae in the Gulf of Tortugas suggested consistent patterns in taxonomic composition, spatiotemporal variability, and environmental modulation. Assemblages were dominated by *Cetengraulis mysticetus*, while recurrent contributions from Sciaenidae, Haemulidae, Gerreidae, and Carangidae pointed to estuarine–shelf linkages. Diversity indices indicated variability among campaigns. Post–La Niña years tended to show higher richness and more balanced assemblages, whereas El Niño phases coincided with reduced diversity and strong dominance. Local drivers—transparency, chlorophyll a, zooplankton biomass, and pH—appeared to influence larval distribution, suggesting coupling between productivity, water quality, and circulation.

**Figure 8:**
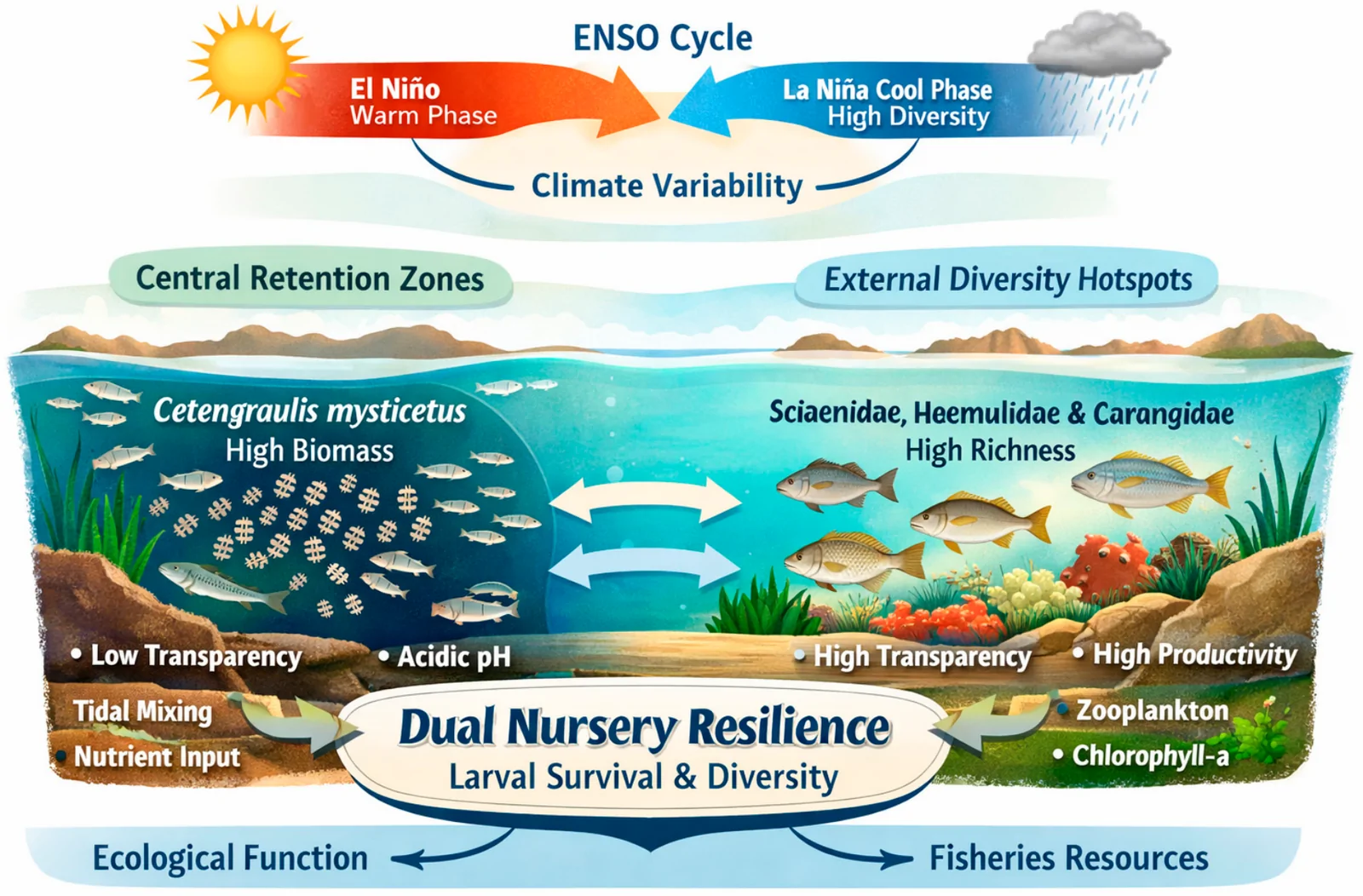
Conceptual model showing how ENSO variability interacts with local drivers to shape nursery function. Central retention zones sustain anchovy biomass, while peripheral dispersive zones act as biodiversity refuges. Together, these complementary habitats strengthen ecological function and fisheries resources, exemplifying pathways toward resilient coastal ecosystems in the Colombian Eastern Tropical Pacific. Source: Copilot Microsoft^®^ 365 Personal, modified by the authors.

Ecologically, the Gulf of Tortugas appeared to function as a dual nursery system. Central retention zones sustained anchovy biomass, while peripheral dispersive zones acted as diversity hotspots. The prevalence of preflexion stages and deviations from neutrality suggested that circulation and retention played a role in structuring nursery function. ENSO variability interacted with local processes, amplifying or dampening their effects depending on climatic phase. Collectively, these observations positioned the Gulf of Tortugas as both a biomass reservoir and a biodiversity refuge, highlighting its potential role as a hotspot of resilience and ecological stability within the Colombian Eastern Tropical Pacific (Figure 8).

## 4. Discussion

Our results provided a cohesive picture of nursery function in the Gulf of Tortugas, integrating taxonomic composition, circulation dynamics, local biophysical drivers, and ENSO variability. This integrative perspective highlights how ecological processes in the region are not governed by a single factor but rather emerge from the interaction of biological assemblages, physical circulation, and climatic oscillations.

### 4.1. Nursery Function in the Context of Coastal Connectivity

The Gulf of Tortugas exemplifies how nursery function emerges from the interaction of circulation, local biophysical drivers, and climatic variability, embedded within a broader framework of coastal connectivity. The dominance of *Cetengraulis mysticetus* across campaigns reflected the importance of estuarine–shelf linkages, where retention zones concentrated biomass of key fisheries species. At the same time, the recurrent presence of families such as Carangidae, Sciaenidae, and Gobiidae in peripheral dispersive zones highlighted the role of biodiversity refuges in sustaining taxonomic breadth. This duality aligned with nursery theory, which emphasizes biomass production and diversity preservation as complementary mechanisms underpinning resilience [37,80,81].

Our circulation estimates represent average drift velocities derived from two GPS records per deployment. While this approach is suitable for identifying broad circulation patterns, it does not capture fine-scale meandering or short-term variability. Future studies should incorporate continuous GPS tracking to improve temporal resolution and trajectory detail. Nevertheless, circulation processes provided the physical template for larval distribution. Inflow introduced biomass, retention favored aggregation, and dispersive pathways facilitated export. Local productivity and water clarity acted as bottom-up drivers, reinforcing assemblage structure under favorable conditions and constraining diversity under stress. ENSO variability amplified these dynamics, suppressing resilience during El Niño and promoting recovery during La Niña. These tendencies underscored the vulnerability of nursery systems to global climatic oscillations [82,83,84].

From a connectivity perspective, the Gulf functioned not as an isolated nursery but as a node within a larger coastal network. Retention zones ensured biomass concentration is critical for local recruitment, while dispersive pathways exported larvae into oceanic assemblages, contributing to regional diversity. Comparable ecological functions have been documented in Bahía Magdalena, Mexico [28,29], Chilean fjords [31], Yangtze Estuary [85] and South African estuaries [24]. These parallels situated the Gulf of Tortugas within a broader Eastern Tropical Pacific nursery network.

### 4.2. Resilience and Vulnerability to Climatic Oscillations

The Gulf of Tortugas nursery system demonstrated both resilience and vulnerability in response to ENSO variability. El Niño phases consistently suppressed diversity, with *Cetengraulis mysticetus* accounting for more than 60% of total abundance during these events. These conditions coincided with weakened inflow, reduced retention efficiency, and diminished productivity. Together, they underscored how climatic oscillations can destabilize nursery function by concentrating biomass into a few dominant taxa [27,30,86,87,88]. In contrast, La Niña phases promoted recovery. Richness increased by 25–30% compared to El Niño years, and Shannon diversity (H′) values nearly doubled. Peripheral dispersive zones played a buffering role, supporting families such as Carangidae, Sciaenidae, and Gobiidae. These areas maintained ecological heterogeneity even under variable hydrographic conditions. Caswell’s neutral model statistic (V) approached neutrality during La Niña campaigns, suggesting more balanced recruitment and stronger stochastic contributions to assemblage structure. Comparable ENSO-related impacts have been documented in the South China Sea [33], the Mediterranean [19,20], and other ocean basins, reinforcing the global relevance of these dynamics.

Low-diversity, high-dominance assemblages occurred under both reduced retention during El Niño and enhanced retention during La Niña, reflecting distinct, context-dependent mechanisms of stratification and biomass concentration. Vertical and horizontal stratification interacted in shaping larval assemblage dynamics in the Gulf of Tortugas. Elevated Brunt–Väisälä frequency (N^2^) during El Niño phases suggested stronger vertical stability, coinciding with deeper thermoclines, reduced nutrient flux, and lower larval biomass. Simultaneously, flattened horizontal gradients, expressed through potential energy anomaly (Φ), indicated weakened inflow and reduced retention, conditions that seemed to limit dispersal and favor dominance by a few taxa. In contrast, lower N^2^ values during La Niña reflected weaker vertical stratification and enhanced mixing, while sharper horizontal gradients suggested stronger inflow and clearer retention–dispersal pathways. These combined patterns may have contributed to higher productivity, improved survival, and greater diversity during cooler phases. Overall, they illustrate how local biophysical drivers interact with global climatic oscillations to influence resilience in coastal nursery systems, without indicating a single dominant mechanism [89]. At the same time, they suggest that nursery systems may absorb climatic shocks [90], while their stability appears to depend on the balance between aggregation and diversity [91].

### 4.3. Implications for Fisheries Management and Conservation

The dual nursery function identified in the Gulf of Tortugas provided insights into how resilience in marine ecosystems can be sustained under changing ocean conditions. Central retention zones, dominated by *Cetengraulis mysticetus*, acted as biomass reservoirs that secured recruitment of key fisheries species. Their vulnerability to climatic suppression during El Niño events highlighted the need for adaptive, climate-responsive management strategies [9,45]. Protecting these retention areas is essential to maintain biomass production, yet management frameworks must anticipate periods of reduced resilience when dominance intensifies and diversity declines.

Peripheral dispersive zones, in contrast, functioned as biodiversity refuges. They supported a wider array of families and enhanced ecological heterogeneity. Their role in buffering against climatic oscillations underscored the importance of conserving habitat mosaics rather than focusing solely on biomass-rich areas [92,93]. By maintaining taxonomic breadth, these zones contributed to long-term resilience and reduced dependence on a single dominant taxon, thereby strengthening ecosystem functioning under variable conditions.

From a conservation perspective, the Gulf of Tortugas should be recognized as a critical nursery system within the Colombian Pacific. Ecological stability depended on the complementary roles of retention and dispersive habitats. Integrating circulation dynamics, local biophysical drivers, and ENSO variability into ecosystem-based fisheries management would allow for more robust strategies that safeguard both biomass and biodiversity. This approach directly supported the goals of resilient seas, emphasizing nursery habitats as foundational components of marine biodiversity and ecological functioning in a changing ocean [94].

## 5. Conclusions

Nursery function in the Gulf of Tortugas appeared to be shaped by taxonomic composition, circulation dynamics, local biophysical drivers, and ENSO variability. The dominance of *Cetengraulis mysticetus*, together with recurrent contributions from estuarine–shelf families, suggested expression through both biomass concentration and taxonomic diversity. Circulation provided the physical template for larval distribution, while bottom-up drivers such as chlorophyll a and zooplankton biomass influenced assemblage structure. ENSO phases modulated these dynamics, with El Niño generally linked to reduced diversity and La Niña to partial recovery, highlighting both vulnerability and adaptive capacity to climatic oscillations. Although differences in sampling months and station numbers may have influenced assemblage composition, pooling by locality and standardizing metrics minimized these effects, allowing interannual patterns to be interpreted in the context of broader ENSO dynamics.

Central retention zones acted as biomass reservoirs dominated by a few species. Peripheral dispersive zones served as biodiversity refuges with higher taxonomic richness. These contrasting habitats complemented each other within the Gulf of Tortugas. Retention zones concentrated biomass, supporting recruitment potential. Dispersive zones promoted diversity export, enhancing ecological connectivity. Together, they appeared to balance aggregation and dispersal processes. This balance contributed to resilience by buffering against environmental variability. It also supported ecological stability across seasonal and interannual scales. Such complementary roles positioned the Gulf of Tortugas as a key nursery system in the Colombian Pacific. Protecting both habitat types may be essential for sustaining biodiversity and fisheries productivity.

Adaptive, climate-responsive management in the Gulf of Tortugas should include delineation of retention and dispersive zones, seasonal closures linked to El Niño forecasts, and intensified monitoring during transitional ENSO phases, thereby operationalizing ecosystem-based fisheries management in a spatially and temporally explicit manner. Safeguarding both habitat types is essential to sustaining fisheries productivity and biodiversity. Integrating local biophysical modulation with global climatic influences into ecosystem-based frameworks enables strategies that anticipate variability and reinforce resilience. In the broader context of Towards Resilient Seas, the Gulf of Tortugas exemplifies how protecting nursery habitats strengthens marine biodiversity and ecological functioning, offering pathways toward resilient coastal ecosystems in a changing ocean.

## Figures and Tables

**Table 1:** Univariate diversity indices across sampling campaigns in the Gulf of Tortugas, including species richness (S), larval density (N, ind/m^3^), Pielou’s evenness (J’), Shannon–Wiener diversity (H’), and Hill numbers (N1, N2). Results from Caswell’s neutral model are also shown, including number of individuals (n), number of species (S), expected diversity under neutrality (E[H’]), standard deviation (SD), and Caswell’s statistic (V), providing a comparative assessment of assemblage structure and departures from neutral expectations.

Campaigns	N	S	J	N1	N2	H’	E[H’]	SD[H’]	V(N.D.)
2010-03	2089	18	0.81	10.48	8.03	2.35	1.73	0.30	2.06
2011-04	3901	31	0.49	5.31	3.11	1.67	2.20	0.26	−2.03
2012-05	5409	60	0.43	5.78	2.33	1.76	2.87	0.20	−5.58
2014-05	16,471	43	0.64	11.19	6.21	2.42	2.34	0.26	0.29
2015-03	13,296	46	0.71	15.06	6.67	2.71	2.44	0.25	1.11
2017-10	78,494	48	0.36	4.03	1.90	1.39	2.27	0.27	−3.24
2019-03	46,419	57	0.75	20.6	10.46	3.02	2.51	0.25	2.10
2019-08	37,224	42	0.71	13.95	8.66	2.63	2.22	0.27	1.52
2020-02	11,786	24	0.83	14.05	11.02	2.64	1.79	0.31	2.74

**Table 2:** Results of the PERMANOVA analyses applied to ichthyoplankton assemblages and environmental variables in the Gulf of Tortugas. Factors evaluated include Campaign, Station, and Zone for both larval and environmental matrices. Reported statistics are degrees of freedom (df), sum of squares (SS), mean squares (MS), Pseudo-F values, permutation-based significance (*P*(perm)), and number of permutations (perms).

Source	df	SS	MS	Pseudo-F	*P* (Perm)	Perms
Ichthyoplankton component						
Campaign	8	84,510	10,564	4.14	0.001	996
Station	14	58,537	4181.2	1.41	0.002	996
Zone	2	14,088	7043.9	2.31	0.001	999
Physical, Chemical, and Biological Components						
Campaign	8	287.82	35.978	5.18	0.001	998
Station	14	315.26	22.519	3.18	0.001	993
Zone	2	199.37	99.685	13.53	0.001	997

## Data Availability

The data supporting the findings of this study are available from the corresponding author upon reasonable request. Raw ichthyoplankton records and processed datasets generated under Permit Res-1070 (issued by the National Authority of Environmental Licenses, ANLA) are archived by the Oceanographic Sciences Research Group at Universidad del Valle. Vouchers of fish larvae collected during the study were deposited in the Zoological Practices Collection of Universidad del Valle. Summary tables are provided within the article and its Supplementary Materials.

## Supplementary Materials

The following supporting information can be downloaded at https://www.mdpi.com/article/10.3390/life16091468/s1, Table S1. Oceanographic parameters recorded at sampling stations during each survey campaign (*n* = number of stations per campaign). Variables include depth (Z, m), temperature (T, °C), salinity (S, PSU), dissolved oxygen (OD, mg·m^−3^), pH, water transparency (Tran, m), and potential energy anomaly (Φ). Values are presented for each station across campaigns conducted between 2010 and 2020. Table S2: Taxonomic richness and relative abundance of fish larvae in the Tortugas Gulf, Colombian Pacific (Eastern Tropical Pacific). RA: Relative abundance (%). CI = Commercial interest. Orders and Families are presented in bold. N/N: Unidentified. Table S3: Biological parameters recorded at sampling stations during each survey campaign. Variables include taxa richness (R), abundance of fish larvae (N, individuals), Shannon diversity index (H), Pielou’s evenness index (J), chlorophyll a concentration (Clo a, mg·m^−3^), and zoo-plankton biomass (B, mg·m^−3^). Values are presented for each station across campaigns conducted between 2010 and 2020.

## References

[1] Rodríguez J., Rubín J. (1991). El ictioplancton y la biomasa del zooplancton en aguas del sur de Galicia, en abril 1987. Bol. Inst. Esp. Oceanogr..

[2] da Silva J.C., Bialetzki A. (2019). Early life history of fishes and zooplankton availability in a Neotropical floodplain: Predator–prey functional relationships. J. Plankton Res..

[3] Lomartire S., Marques J.C., Gonçalves A.M. (2021). The key role of zooplankton in ecosystem services: A perspective of interaction between zooplankton and fish recruitment. Ecol. Indic..

[4] Quah W.C., Chew L.L., Chong V.C., Chu C., Teoh C.Y., Ooi A.L. (2022). Does structural change in the zooplankton community affect larval fish feeding in anthropogenically disturbed tropical waters?. Environ. Biol. Fish..

[5] Swalethorp R., Landry M.R., Semmens B.X., Ohman M.D., Aluwihare L., Chargualaf D., Thompson A.R. (2023). Anchovy boom and bust linked to trophic shifts in larval diet. Nat. Commun..

[6] Köster F.W., Hinrichsen H., St. John M.A., Schnack D., MacKenzie B.R., Tomkiewicz J., Plikshs M. (2001). Developing Baltic cod recruitment models. II. Incorporation of environmental variability and species interaction. Can. J. Fish. Aquat. Sci..

[7] Frederiksen M., Edwards M., Richardson A.J., Halliday N.C., Wanless S. (2006). From plankton to top predators: Bottom-up control of a marine food web across four trophic levels. J. Anim. Ecol..

[8] Montagnes D.J.S., Dower J.F., Figueiredo G.M. (2010). The protozooplankton–ichthyoplankton trophic link: An overlooked aspect of aquatic food webs. J. Eukaryot. Microbiol..

[9] Walsh H.J., Richardson D.E., Marancik K.E., Hare J.A. (2015). Long-term changes in the distributions of larval and adult fish in the northeast US shelf ecosystem. PLoS ONE.

[10] Kallasvuo M., Vanhatalo J., Veneranta L. (2017). Modeling the spatial distribution of larval fish abundance provides essential information for management. Can. J. Fish. Aquat. Sci..

[11] Álvarez P., Garcia D., Cotano U. (2023). Investigating the applicability of ichthyoplanktonic indices in better understanding the dynamics of the northern stock of the population of Atlantic hake *Merluccius merluccius* (L.). Fishes.

[12] Lasker R. (1981). Factors contributing to variable recruitment of the northern anchovy (*Engraulis mordax*) in the California Current: Contrasting years, 1975 through 1978. Rapp. Procès-Verbaux Réun. Cons. Int. Explor. Mer..

[13] Pritt J.J., Roseman E.F., Ross J.E., DeBruyne R.L. (2015). Using larval fish community structure to guide long-term monitoring of fish spawning activity. N. Am. J. Fish. Manag..

[14] Legrand T., Di Franco A., Ser-Giacomi E., Caló A., Rossi V. (2019). A multidisciplinary analytical framework to delineate spawning areas and quantify larval dispersal in coastal fish. Mar. Environ. Res..

[15] Guyah N., Webber M., Prospere K. (2021). An assessment of the larval fish diversity within a coastal marine reserve. Reg. Stud. Mar. Sci..

[16] Wang D., Yu J., Lin Z., Chen P. (2023). Spatial–temporal distribution of fish larvae in the Pearl River estuary based on habitat suitability index model. Biology.

[17] Giraldo A., Valencia B., Martínez T.I., Ramírez D.G., Valencia B., Giraldo A. (2008). Condiciones oceanográficas en las localidades de La Mina (Punta Cruces) y El Acuario (Cabo Marzo), departamento del Chocó, zona norte de la costa Pacífica colombiana. Caracterización Ecológica de los Arrecifes Coralinos y Bosques de Manglar en Cabo Marzo, Zona Norte del Litoral Pacífico Colombiano: Estructura, Composición y Diversidad y Fauna Asociada.

[18] López-Chávez O., Aceves-Medina G., Saldierna-Martínez R.J., Jiménez-Rosenberg S.P., Murad-Serrano J.P., Marín-Gutiérrez Á., Hernández-Hernández O. (2012). Changes in species composition and abundance of fish larvae from the Gulf of Tehuantepec, Mexico. CICIMAR Oceánides.

[19] Basilone G., Bonanno A., Patti B., Mazzola S., Barra M., Cuttitta A., McBride R. (2013). Spawning site selection by European anchovy (*Engraulis encrasicolus*) in relation to oceanographic conditions in the Strait of Sicily. Fish. Oceanogr..

[20] Giannoulaki M., Iglesias M., Tugores M.P., Bonanno A., Patti B., De Felice A., Leonori I., Bigot J.L., Ticina V., Pyrounaki M.M. (2013). Characterizing the potential habitat of European anchovy *Engraulis encrasicolus* in the Mediterranean Sea at different life stages. Fish. Oceanogr..

[21] Chiappa-Carrara X., Sanvicente-Añorve L., Monreal-Gómez A., Salas de León D. (2003). Ichthyoplankton distribution as an indicator of hydrodynamic conditions of a lagoon system in the Mexican Caribbean. J. Plankton Res..

[22] Lee O., Nash R., Danilowicz B. (2005). Small-scale spatio-temporal variability in ichthyoplankton and zooplankton distribution in relation to a tidal-mixing front in the Irish Sea. ICES J. Mar. Sci..

[23] Campbell R., Díaz F., Hu Z., Doglioli A., Petrenko A., Dekeyser I. (2013). Nutrients and plankton spatial distributions induced by a coastal eddy in the Gulf of Lion: Insights from a numerical model. Prog. Oceanogr..

[24] Strydom N.A., Booth A.J., McLachlan A. (2014). Occurrence of larval fishes in a rocky shore-associated nursery area in temperate South Africa, with emphasis on temperature-related growth in dominant Sparidae. Afr. J. Mar. Sci..

[25] Cotrim-Marques S., Miranda-Azeiteiro U., Marques J.C., Neto J.M., Pardal M.A. (2006). Zooplankton and ichthyoplankton communities in a temperate estuary: Spatial and temporal patterns. J. Plankton Res..

[26] Militelli M.I., Rodrigues K.A., Cortés F., Macchi G.J. (2013). Influencia de los factores ambientales en el desove de los esciénidos en la zona costera de Buenos Aires, Argentina. Cienc. Mar..

[27] Tsai C.F., Chen P.Y., Chen C.P., Lee M.A., Shiah G.Y., Lee K.T. (2003). Fluctuation in abundance of larval anchovy and environmental conditions in coastal waters off south-western Taiwan as associated with the El Niño–Southern Oscillation. Fish. Oceanogr..

[28] Sánchez-Velasco L., Green-Ruíz C.C., Juárez-Olvera C., Cervantes M.J., Barreiro-Güemes T., Meave del Castillo M.E., Signoret Poillon M., Figueroa Torres M.G. (2003). Estado del conocimiento del ictioplancton del Pacífico noroccidental mexicano. Planctología Mexicana.

[29] Sánchez-Velasco L., Avalos-García C., Rentería-Cano M., Shirasago B. (2004). Fish larvae abundance and distribution in the central Gulf of California during strong environmental changes (1997–1998 El Niño and 1998–1999 La Niña). Deep Sea Res. Part II.

[30] Funes-Rodríguez R., Flores-Coto C., Esquivel-Herrera A., Fernández-Alamo M.A., García-Gasca A. (2002). Larval fish community structure along the west coast of Baja California during and after the El Niño event (1983). Bol. Cienc. Mar..

[31] Landaeta M.F., Schrebler K., Bustos C.A., Letelier J., Balbontín F. (2009). Temporal fluctuations of nearshore ichthyoplankton off Valparaíso, central Chile, during the ENSO cycle 1997–2000. Rev. Biol. Mar. Oceanogr..

[32] Martínez-Aguilar T., Giraldo A., Rodríguez-Rubio E. (2010). Ictioplancton en la zona costera del Pacífico colombiano durante la fase terminal de El Niño 2006–2007. Lat. Am. J. Aquat. Res..

[33] Kim Y.Y., Qu T., Jensen T., Miyama T., Mitsudera H., Kang H.W., Ishida A. (2004). Seasonal and interannual variations of the North Equatorial Current bifurcation in a high-resolution OGCM. J. Geophys. Res. Oceans.

[34] Franco-Gordo C., Godínez-Domínguez E. (2026). Seasonal Hydrography and ENSO Variability Shape Ichthyoplankton Assemblage Structure in the Central Mexican Pacific. Diversity.

[35] Vollrath S.R., Tanner S.E., Reis-Santos P., Possamai B., Grimm A.M., Gillanders B.M., Garcia A.M. (2023). Complex interactions of ENSO and local conditions buffer the poleward shift of migratory fish in a subtropical seascape. Sci. Total Environ..

[36] Valencia B., Rivera-Gómez M., Jerez-Guerrero M., Rondón-Ramos M., Giraldo A. (2024). Temporal and spatial variability of ichthyoplankton assemblages in the Eastern Tropical Pacific off Colombia. Cont. Shelf Res..

[37] Nagelkerken I., Sheaves M., Baker R., Connolly R.M. (2015). The seascape nursery: A novel spatial approach to identify and manage nurseries for coastal marine fauna. Fish Fish..

[38] Whitfield A.K. (2020). Littoral habitats as major nursery areas for fish species in estuaries: A reinforcement of the reduced predation paradigm. Mar. Ecol. Prog. Ser..

[39] Guerreiro M.A., Martinho F., Baptista J., Costa F., Pardal M.Á., Primo A.L. (2021). Function of estuaries and coastal areas as nursery grounds for marine fish early life stages. Mar. Environ. Res..

[40] Zhang H., Li Y., Jiang C. (2022). Biological and ecological studies on marine ichthyoplankton. Front. Mar. Sci..

[41] Franco-Gordo C., Godínez-Domínguez E., Suárez-Morales E. (2002). Larval fish assemblages in waters off the central Pacific coast of Mexico. J. Plankton Res..

[42] León-Chávez C.A., Sánchez-Velasco L., Beier E., Lavín M.F., Godínez V.M., Färber-Lorda J. (2010). Larval fish assemblages and circulation in the Eastern Tropical Pacific in autumn and winter. J. Plankton Res..

[43] León-Chávez C.A., Beier E., Sánchez-Velasco L., Barton E.D., Godínez V.M. (2015). Role of circulation scales and water mass distributions on larval fish habitats in the Eastern Tropical Pacific off Mexico. J. Geophys. Res. Oceans.

[44] Urias-Leyva H., Aceves-Medina G., Avendaño-Ibarra R., Saldierna-Martínez R.J., Gómez-Gutiérrez J., Robinson C.J. (2018). Regionalization in the distribution of larval fish assemblages during winter and autumn in the Gulf of California. Lat. Am. J. Aquat. Res..

[45] Amezcua F., Rodríguez-Preciado J.A., Calderón-Pérez A., Rendón-Rodríguez S., Green L., Chazarreta C.J. (2020). Larval fish assemblages in nearshore waters of southeast Gulf of California: Vertical and temporal patterns. Neotrop. Ichthyol..

[46] Eisele M.H., Madrigal-Mora S., Espinoza M. (2021). Drivers of reef fish assemblages in an upwelling region from the Eastern Tropical Pacific Ocean. J. Fish Biol..

[47] Gallego-Zerrato J.J., Cuellar A., Giraldo A. (2025). Ichthyoplankton composition and environmental drivers in the Sanquianga Tapaje Estuarine System, Eastern Tropical Pacific. Diversity.

[48] Jiménez-Quiroz M.C., Barron-Barraza F.J., Cervantes-Duarte R., Funes-Rodriguez R., Gonzalez-Armas R. (2024). Differences in the impact of intense ENSO+ in Bahía Magdalena (SW of Baja California, Mexico) in the context of climate change. Reg. Stud. Mar. Sci..

[49] Funes-Rodríguez R., Leal-Espinoza J.D., Hinojosa-Medina A., Hernández-Rivas M.E., Flores-Coto C., Funes-Rodríguez R., Gómez-Gutiérrez J., Palomares-García R. (2007). Composición, distribución y abundancia de larvas de peces en Bahía Magdalena. Estudios ecológicos en Bahía Magdalena.

[50] Jiménez-Quiroz M.C., Cervantes-Duarte R., Funes-Rodríguez R., Barón-Campis S.A., García-Romero F.J., Hernández-Trujillo S., Hernández-Becerril D.U., González-Armas R., Martell-Dubois R., Cerdeira-Estrada S. (2019). Impact of “The Blob” and “El Niño” in the SW Baja California Peninsula: Plankton and environmental variability of Bahía Magdalena. Front. Mar. Sci..

[51] Swalethorp R., Malanski E., Agersted M.D., Nielsen T.G., Munk P. (2015). Structuring of zooplankton and fish larvae assemblages in a freshwater-influenced Greenlandic fjord: Influence from hydrography and prey availability. J. Plankton Res..

[52] Mocuba J., Leitão F., Teodósio M.A. (2023). The Diversity of Fish Larvae in the Bons Sinais Estuary (Mozambique) and Its Role as a Nursery to Marine Fish Resources. Diversity.

[53] Arevalo E., Cabral H.N., Villeneuve B., Possémé C., Lepage M. (2023). Fish larvae dynamics in temperate estuaries: A review on processes, patterns and factors that determine recruitment. Fish Fish..

[54] de Lima L.G., Araújo F.G., Macário B.S., Pessanha A.L.M. (2024). Larval fish assemblages in selected Brazilian estuaries: Species–environment relationships under different anthropogenic influences. Mar. Pollut. Bull..

[55] Sodré D., Costa A., Silva E., Pereira L., Costa R. (2026). Determining the Diversity and Environmental Structuring of Fish Larvae in an Amazonian Coastal Protected Estuary. Oceans.

[56] Cheminée A., Le Direach L., Rouanet E., Astruch P., Goujard A., Blanfuné A., Bonhomme D., Chassaing L., Jouvenel J.-Y., Ruitton S. (2021). All shallow coastal habitats matter as nurseries for Mediterranean juvenile fish. Sci. Rep..

[57] Sun Y.H., Wang Y.C., Hsu L.H., Meng P.J., Hsieh H.Y. (2026). Upwelling as a nursery for fish larvae: Summer observations from the Taiwan Bank. Reg. Stud. Mar. Sci..

[58] Tiedemann M., Fock H.O., Brehmer P., Döring J., Möllmann C. (2017). Does upwelling intensity determine larval fish habitats in upwelling ecosystems? The case of Senegal and Mauritania. Fish. Oceanogr..

[59] Echeverri C., Bergamín H. (1982). Estudio Preliminar de la Taxonomía y Distribución de Larvas de Peces en la Bahía de Buenaventura. Bachelor’s Thesis.

[60] Rueda-Montenegro C., Caraballo P. (1984). Composición, Distribución y Abundancia del Ictioplancton en el Pacífico Colombiano, Nov–Dic 1982. Bachelor’s Thesis.

[61] Jiménez S., Arboleda E.A. (1991). Distribución y abundancia de larvas y huevos de peces durante el Crucero Pacífico XIII ERFEN X, noviembre de 1988. Bol. Cient. CCCP.

[62] Beltrán B., Ramos G., Escobar J., Tovar J. (1994). Distribución y abundancia de huevos y larvas de *Opisthonema* spp. y *Cetengraulis mysticetus* (Pisces: Clupeiformes) en el Pacífico colombiano durante enero de 1993. Bol. Cient. INPA.

[63] Moreno A. (1995). Descripción, Distribución y Abundancia de Larvas y Postlarvas de Sciaenidae (Pisces, Perciformes) en el Pacífico Colombiano Durante 1991. Bachelor’s Thesis.

[64] Escarria E., Beltrán B., Giraldo A., Galvis R. (2005). Composición, distribución y abundancia del ictioplancton en la cuenca del océano Pacífico colombiano durante septiembre de 2003. Bol. Cient. CCCP.

[65] Escarria E., Beltrán-León B., Giraldo A. (2006). Ictioplancton superficial de la cuenca del océano Pacífico colombiano (septiembre 2003). Investig. Mar..

[66] Escarria E., Beltrán B., Giraldo A., Zapata F. (2007). Ichthyoplankton in the National Natural Park Isla Gorgona (Pacific Ocean of Colombia) during September 2005. Investig. Mar..

[67] Giraldo A. (2020). Ictioplancton de Cabo Manglares (Distrito Nacional de Manejo Integrado Cabo Manglares, Bajo Mira y Frontera), Nariño, Colombia. Bol. Cient. Mus. Hist. Nat..

[68] Rondón M., Giraldo A. (2025). Taxonomic richness, diversity, temporal variation, and ecological aspects of fish larvae in the Gulf of Tribugá, Colombia. Rev. Biol. Trop..

[69] Calle-Bonilla I.C., Giraldo A. (2026). Larval fish assemblage dynamics in response to ENSO events in the Eastern Tropical Pacific: Evidence from Gorgona Island, Colombia. Reg. Stud. Mar. Sci..

[70] Castellanos-Galindo G.A., Zapata Padilla L.A., Salas S., Barragán-Paladines M., Chuenpagdee R. (2019). Small-scale fisheries on the Pacific coast of Colombia: Historical context, current situation, and future challenges. Viability and Sustainability of Small-Scale Fisheries in Latin America and The Caribbean.

[71] Beltrán-León B., Ríos R.H. (2000). Estadíos Tempranos de Peces del Pacífico Colombiano.

[72] Moser H.G. (1996). The Early Stages of Fishes in the California Current Region.

[73] Lorenzen C.J. (1967). Determination of chlorophyll and pheo-pigments: Spectrophotometric equations. Limnol. Oceanogr..

[74] Postel L., Fock H., Hagen W., Harris R.P., Wiebe P.H., Lenz J., Skjoldal H.R., Huntley M. (2000). Biomass and abundance. ICES Zooplankton Methodology Manual.

[75] Boucher G., Lambshead J.D. (1995). Ecological biodiversity of marine nematodes in samples from temperate, tropical, and deep-sea regions. Conserv. Biol..

[76] Clarke K.R., Warwick R.M. (2001). Change in Marine Communities: An Approach to Statistical Analysis and Interpretation.

[77] Anderson M.J. (2001). A new method for non-parametric multivariate analysis of variance. Austral Ecol..

[78] Anderson M.J., Gorley R.N., Clarke K.R. (2008). PERMANOVA+ for PRIMER: Guide to Software and Statistical Methods.

[79] Anderson M.J. (2005). PERMANOVA: A FORTRAN Computer Program for Permutational Multivariate Analysis of Variance.

[80] Sheaves M., Baker R., Nagelkerken I., Connolly R.M. (2015). True value of estuarine and coastal nurseries for fish: Incorporating complexity and dynamics. Estuaries Coasts.

[81] De Battisti D. (2021). The resilience of coastal ecosystems: A functional trait-based perspective. J. Ecol..

[82] Martínez Arroyo A., Manzanilla Naim S., Zavala Hidalgo J. (2011). Vulnerability to climate change of marine and coastal fisheries in México. Atmósfera.

[83] Cavraro F., Anelli Monti M., Matić-Skoko S., Caccin A., Pranovi F. (2023). Vulnerability of the Small-Scale Fishery to Climate Changes in the Northern-Central Adriatic Sea (Mediterranean Sea). Fishes.

[84] Makame O.M., Salum L.A. (2021). Vulnerability of fishing and fisheries sector to climate change and non-climate risks as perceived by fishermen in Zanzibar coastal villages. Carnets Rech. l’Océan Indien.

[85] Zhang H., Xian W., Liu S. (2016). Autumn ichthyoplankton assemblage in the Yangtze Estuary shaped by environmental factors. PeerJ.

[86] Attrill M.J., Power M. (2002). Climatic influence on a marine fish assemblage. Nature.

[87] McLean M.J., Mouillot D., Goascoz N., Schlaich I., Auber A. (2019). Functional reorganization of marine fish nurseries under climate warming. Glob. Change Biol..

[88] Colombano D.D., Carlson S.M., Hobbs J.A., Ruhi A. (2022). Four decades of climatic fluctuations and fish recruitment stability across a marine–freshwater gradient. Glob. Change Biol..

[89] O’Leary J.K., Micheli F., Airoldi L., Boch C., De Leo G., Elahi R., Ferretti F., Graham N.A.J., Litvin S.Y., Low N.H. (2017). The resilience of marine ecosystems to climatic disturbances. BioScience.

[90] Fisher M.C., Moore S.K., Jardine S.L., Watson J.R., Samhouri J.F. (2021). Climate shock effects and mediation in fisheries. Proc. Natl. Acad. Sci. USA.

[91] Sterl X., van Leeuwen A., ten Brink H. (2025). Four causes of nursery function degradation and their consequences for declining marine coastal- and estuarine-dependent species. ICES J. Mar. Sci..

[92] Smith J.G., Lopazanski C., Free C.M., Brun J., Anderson C., Carr M.H., Claudet J., Dugan J.E., Eurich J.G., Francis T.B. (2025). Conservation benefits of a large marine protected area network that spans multiple ecosystems. Conserv. Biol..

[93] Rilov G., Mazaris A.D., Stelzenmüller V., Helmuth B., Wahl M., Guy-Haim T., Katsanevakis S. (2019). Adaptive marine conservation planning in the face of climate change: What can we learn from physiological, ecological and genetic studies?. Glob. Ecol. Conserv..

[94] Lefcheck J.S., Hughes B.B., Johnson A.J., Pfirrmann B.W., Rasher D.B., Smyth A.R., Williams B.L., Beck M.W., Orth R.J. (2019). Are coastal habitats important nurseries? A meta-analysis. Conserv. Lett..

