# Supplementary material for: Resilient Seas: Environmental Drivers of Larval Fish Assemblages in the Gulf of Tortugas, Colombian Pacific
Source: Life (Basel). 2026 Sep 2;16(9):1468. doi: 10.3390/life16091468 (PMC13608056; doi:10.3390/life16091468)
Supplement: Supplementary file 1 [file life-16-01468-s001.zip › life-4506680-supplementary.pdf]

Table S1. Oceanographic parameters recorded at sampling stations during each survey campaign (*n* = number of stations per campaign). Variables include depth (Z, m), temperature (T, °C), salinity (S, PSU), dissolved oxygen (OD, mg·m^{-3}), pH, water transparency (Tran, m), and potential energy anomaly (Φ). Values are presented for each station across campaigns conducted between 2010 and 2020.

Campaing	Station	Z	T	S	OD	pH	Tran	Φ
2010-03	1	9.2	29.7	25.3	5.90	8.07	1.0	18.45
n = 9	3	5.8	29.4	27.3	6.16	8.03	3.5	6.37
	5	23.7	29.8	28.2	6.51	8.37	9.0	53.65
	6	23.3	29.2	28.5	6.35	8.23	6.5	29.49
	7	11.8	29.6	27.7	6.39	8.38	4.0	6.39
	8	14.2	29.2	27.1	6.72	8.48	1.0	13.35
	10	43.7	29.0	29.6	6.64	8.36	13.0	340.29
	11	38.5	29.7	28.4	6.34	8.38	9.5	242.20
	12	26.7	29.6	26.8	6.61	8.39	6.5	134.34
2011-04	1	9.2	28.2	27.7	5.64	7.45	1.2	0.47
n = 12	2	5.8	28.3	25.4	5.54	7.11	0.8	6.50
	3	3.9	28.5	26.2	5.70	7.14	0.9	16.10
	4	14.7	28.1	24.7	6.12	7.21	2.5	25.68
	5	24.6	28.4	28.4	6.52	7.39	6.0	101.11
	6	22.7	28.0	27.5	5.6	7.54	2.1	59.08
	7	9.5	28.4	28.4	5.84	7.56	2.6	4.62
	8	23.6	28.4	26.7	6.42	7.61	3.9	75.19
	9	53.1	28.2	27.5	6.2	7.55	3.5	359.15
	10	41.3	28.2	27.3	6.25	7.54	7.4	248.61
	11	41.3	28.1	29.7	6.49	7.4	5.5	200.06
	12	26.8	28.6	24.4	6.24	7.28	3.0	85.69
2012-05	1	9.1	28.0	26.9	5.81	7.72	2.0	13.22
n = 12	3	3.8	28.4	28	5.08	7.55	1.5	1.05
	4	14.1	28.5	27.8	4.36	7.61	3.5	23.03
	5	24.0	28.4	27.8	5.37	7.71	2.0	72.33
	6	23.8	28.1	28.2	6.42	7.45	5.5	81.34
	7	9.1	29.4	29.0	5.99	7.56	2.8	5.99
	8	22.1	29.0	27.4	6.72	7.74	4.7	142.31
	9	59.2	28.6	26.7	6.44	7.68	5.0	383.90
	10	42.1	28.2	28.8	6.57	7.77	8.0	268.43
	11	39.0	28.4	28.6	4.7	7.75	5.0	85.73
	12	27.1	28.2	26.9	3.24	7.69	4.0	35.41
	13	80	28.1	29.3	6.52	7.59	13.3	369.30
2014-05	1	9.2	28.9	24.2	6.44	7.74	1.3	14.39
n = 12	2	9.4	28.7	11.62	6.83	7.95	3.5	4.88
	3	9.2	28.8	10.65	7.62	7.93	3.0	8.68
	4	22.6	28.9	26.09	6.36	8.04	3.0	50.02
	7	9.5	29.4	24.43	6.57	8.09	2.5	7.87
	9	41.1	28.3	27.72	6.97	8.13	3.5	222.20
	10	42.9	28.3	26.33	6.82	8.06	6.5	98.13
	11	52.2	28.1	28.8	5.9	7.99	3.5	335.66
	12	30.6	28.1	28.8	5.8	8.00	3.0	140.65

	13	68.9	28.3	27.52	6.84	8.14	20.0	291.59
	14	73.9	28.9	28.3	6.86	8.14	20.0	323.30
	15	72.0	28.6	26.65	7.03	8.13	6.5	132.72
2015-03	1	8.3	29.5	23.05	6.46	7.72	2.5	21.12
n = 13	2	8.8	28.1	23.82	6.09	7.56	4.0	21.27
	3	7.6	28.4	24.97	4.96	7.61	4.0	6.69
	4	29.9	27.8	26.18	4.95	7.69	7.0	58.81
	5	30.6	28.1	23.95	6.31	7.53	10.0	159.15
	6	28.6	28.3	24.91	6.27	7.63	6.0	113.83
	7	8.5	28.1	25.69	6.86	7.76	4.0	7.57
	8	29.7	28.6	23.79	6.90	7.80	4.0	211.92
	10	43.1	29.2	25.53	6.16	7.76	10.0	276.34
	11	48.7	28.4	24.11	6.02	7.75	12.4	315.66
	12	29.6	28.5	25.3.0	6.37	7.85	5.0	232.04
	13	70.0	28.6	26.21	6.47	7.75	9.0	360.11
	15	77.6	28.3	23.72	6.49	7.79	7.0	431.64
2017-10	1	9.2	28.3	21.36	6.52	8.36	2.0	5.74
n = 15	2	7.5	28.4	20.34	5.74	8.14	0.5	11.31
	3	8.0	28.2	23.63	5.95	8.30	1.6	9.89
	4	20.0	28.2	20.57	6.48	8.21	2.0	79.88
	5	31.6	28.2	23.95	5.53	8.34	7.0	105.04
	6	22.4	27.8	21.57	6.52	8.73	5.7	129.33
	7	10.7	28.9	20.16	6.66	8.39	1.7	35.25
	8	30.9	28.0	19.93	6.83	8.43	2.0	99.87
	9	56.1	27.8	23.12	6.78	8.48	10.9	382.47
	10	39.4	27.6	23.89	6.64	8.33	10.0	113.92
	11	50.4	27.8	25.68	6.60	8.42	5.6	30.13
	12	30.0	28.3	22.68	6.57	8.32	2.0	71.15
	13	80.0	27.7	25.54	6.50	8.34	7.5	374.47
	14	67.0	27.6	24.61	6.55	8.49	12.0	536.09
	15	77.6	27.7	23.14	6.71	8.43	9.0	752.93
2019-03	1	9.4	27.2	24.75	5.13	8.40	3	55.01
n = 15	2	6.3	27.6	23.29	3.34	8.20	1.5	10.14
	3	7.0	27.8	23.69	4.20	7.80	1.5	50.37
	4	21.9	26.4	23.21	4.65	7.40	3.0	90.79
	5	30.5	27.1	25.21	5.08	8.10	5.0	143.97
	6	27.8	26.4	25.02	5.18	7.90	5.0	224.08
	7	10.8	27.4	27.6	5.48	8.20	5.0	64.10
	8	31.9	24.6	28.46	5.12	8.30	5.0	79.52
	9	56.1	27.4	25.33	5.60	8.10	8.0	173.39
	10	40.2	27.7	26.12	5.61	8.30	11.0	142.64
	11	52.7	26.8	26.56	5.60	7.80	11.0	153.37
	12	31.0	26.7	25.93	5.09	7.60	4.0	124.45
	13	71.8	26.6	27.25	5.66	8.00	7.0	238.52
	14	82.0	27.4	25.33	5.60	8.10	13.0	195.07
	15	80.0	27.2	24.63	5.82	8.30	11.0	256.71
2019-08	1	9.4	28.42	21.71	6.04	10.97	1.0	47.84
n = 15	2	9.0	28.42	23.11	6.10	10.42	2.0	18.18
	3	9.8	28.37	23.81	6.45	–	1.8	15.43
	4	24.0	28.5	25.78	6.56	9.53	2.2	117.50
	5	30.8	28.5	24.56	6.57	11.19	1.2	369.50
	6	29.9	28.5	23.05	6.52	10.95	1.0	158.74
	7	8.3	28.2	24.73	6.49	11.52	2.2	31.80

	8	30.2	29.0	24.29	6.96	11.36	2.0	155.65
	9	57.2	28.1	24.91	6.7	11.23	4.0	377.00
	10	42.1	28.2	25.54	6.68	11.31	5.0	168.69
	11	48.6	28.8	26.69	6.68	9.65	2.0	285.87
	12	32.0	28.6	26.91	6.59	9.45	1.5	100.45
	13	69.6	28.2	29.52	6.55	9.58	1.6	548.07
	14	72.4	28.4	27.33	6.68	9.68	2.0	610.63
	15	80.0	28.5	25.26	6.76	11.36	4.4	656.87
2020-02	2	6.7	28.6	27.51	4.92	7.84	1.0	8.94
n = 11	3	7.2	28.2	27.76	7.80	7.86	1.4	10.54
	4	22.5	27.6	24.83	4.50	8.07	2.0	59.25
	5	10.2	23.9	30.07	6.12	7.41	7.0	3.49
	6	31.8	24.9	26.92	4.91	7.30	3.0	250.61
	7	56.9	24.9	29.32	4.32	7.33	5.4	404.35
	8	54.2	26.6	28.33	5.50	7.81	4.0	408.48
	12	31.0	27.3	29.02	5.14	7.85	2.9	201.92
	13	72.5	26.7	30.60	5.08	8.70	16.0	402.31
	14	69.7	26.3	28.92	5.50	7.35	6.4	447.09
	15	76.3	24.8	29.32	4.98	7.35	11.3	469.01

Table S2. Taxonomic richness and relative abundance of fish larvae in the Tortugas Gulf, Colombian Pacific (Eastern Tropical Pacific). RA: Relative abundance (%).CI = Commercial interest. Orders and Families are presented in bold. N/N: Unidentified.

Family/Taxa	RA	Habitat	CI
**Clupeiformes**			
**Engraulidae**	48.81		
Cetengraulis mistycetus	48.61	Coastal pelagic habitat (<200 m)	High
Anchoa sp.	0.04		
Anchoa sp.1	0.07		
Anchoa sp.2	0.09		
**Stomiiformes**			
**Phosichthyidae**	0.04		
Vinciguerria lucetia	0.04	Deep-ocean pelagic habitat (>1000 m)	No
**Gonostomatiidae**	0.01		
Cyclothone pseudopallida	0.01	Deep-ocean pelagic habitat (>1000 m)	No
**Aulopiformes**			
**Synodontidae**	0.23		
Synodus scituliceps	0.20	Marine bottom-dwelling habitat	Medium
Synodus evermanni	0.01	Marine bottom-dwelling habitat	Medium
Synodus sp.	0.01		
**Myctophiformes**			
**Myctophidae**	0.03		
Diogenichthys laternatus	0.01	Open-ocean pelagic habitat (>200 m)	No
Benthosema panamense	0.01	Open-ocean pelagic habitat (>200 m)	No
**Gadiformes**			
**Bregmacerotidae**	0.37		
Bregmaceros bathymaster	0.37	Coastal pelagic habitat (<200 m)	No
**Ophidiiformes**			
**Ophidiidae**	0.14		
Lepophidium sp.	0.14		
**Scombriformes**			
**Scombridae**	0.77		
Scomberomorus sierra	0.19	Coastal pelagic habitat (<200 m)	High
Auxis sp.2	0.09		
Scomber japonicus	0.45	Coastal pelagic habitat (<200 m)	High
Scombridae sp.1	0.04		
**Bramidae**	0.01		
Brama dussumieri	0.01	Coastal pelagic habitat (<200 m)	Low
**Syngnathiformes**			
**Fistulariidae**	0.09		
Fistularia corneta	0.09	Coastal pelagic habitat (<200 m)	No
**Syngnathidae**	0.03		
Syngnathus sp.	0.03		
**Gobiiformes**			
**Gobiidae**	13.04		
Gobiidae sp.1	0.33		
Ilypnus gilberti	0.05	Marine bottom-dwelling habitat	No
Microgobius sp.	0.57		
Lepidogobius sp.	0.03		

Gobiidae sp.4	5.16		
Gobiidae sp.2	0.03		
Clarckichthys bilineatus	0.18	Reef-associated habitat	No
Lythrypnus sp.	1.82		
Gobulus sp.	1.41		
Gobiidae sp.7	0.14		
Gobiidae sp.8	0.16		
Gobiidae sp. 9	0.09		
Gobiidae sp.10	0.01		
Gobiidae sp.11	0.07		
Gobiidae sp.12	0.01		
Gobiidae sp. 6	0.01		
**Gobiidae**	2.37		
Bathygobius sp.	0.01		
Bollmannia sp.	0.43		
Tigrigobius sp.	0.15		
**Eleotridae**	1.97		
Eleotridae sp.	1.95		
Gobiomorus sp.	0.01		
**Carangiformes**			
**Centropomidae**	0.30		
Centropomus sp.	0.30		
**Carangidae**	1.50		
Oligoplites saurus	0.35	Coastal pelagic habitat (<200 m)	High
Seriola sp.	0.19		
Oligoplites sp.	0.08		
Trachurus symmetricus	0.04	Pelagic habitat	Low
Caranx caballus	0.03	Coastal pelagic habitat (<200 m)	High
Caranx sp.	0.12		
Caranx sp.3	0.04		
Seriola lalandi	0.07	Open-ocean pelagic habitat (>200 m)	Low
Chloroscombrus orqueta	0.30	Benthopelagic habitat	Medium
Selene peruviana	0.03	Open-ocean pelagic habitat (>200 m)	Medium
Selar crumenophthalmus	0.15	Reef-associated habitat	Medium
Decapterus sp.	0.09		
Caranx sp.1	0.01		
**Polynemidae**	0.03		
Polydactylus opercularis	0.03		
**Coryphaenidae**	0.22		
Coryphaena hippurus	0.22	Pelagic habitat	High
**Serranidae**	0.35		
Serranus sp.	0.35		
**Sphyraenidae**	0.11		
Sphyraena ensis	0.08	Coastal pelagic habitat (<200 m)	High
Sphyraena sp.1	0.03		
**Pleuronectiformes**			
**Achiridae**	0.83		
Achirus mazatlanus	0.69	Brackish bottom-dwelling habitat	Low
Achirus klunzingeri	0.07	Brackish bottom-dwelling habitat	Low
Trinectes sp.1	0.04		
Trinectes sp.2	0.03		
**Cyclopsettidae**	0.14		
Citharichthys gilberti	0.14	Marine bottom-dwelling habitat	Low

**Bothidae**	0.08		
Bothidae sp.	0.08		
**Paralichthyidae**	0.20		
Paralichthyidae sp.1	0.20		
**Insertae sedis in Ovalentaria**			
**Pomacentridae**	0.47		
Pomacentridae sp.	0.41		
Stegastes sp.	0.05		
Chromis sp.	0.01		
**Opistognathidae**	0.12		
Opistognathus sp.	0.12		
**Mugiliformes**			
**Mugilidae**	0.14		
Mugil setosus	0.11		
Mugil cephalus	0.03	Benthopelagic habitat	High
**Gobiesociformes**			
**Gobiesocidae**	0.96		
Gobiesox sp.6	0.56		
Gobiesox sp.3	0.19		
Gobiesox sp.1	0.14		
Gobiesocidae sp.	0.03		
Gobiesox sp.2	0.05		
**Bleniiformes**			
**Labrisomidae**	0.11		
Labrisomus sp.	0.09		
Labrisomidae sp.	0.01		
**Blenniidae**	0.09		
Hypsoblennius brevipinnis	0.09	Reef-associated habitat	No
**Insertae sedis in Eupercaria**			
**Sciaenidae**	18.61		
Menticirrhus sp.1	0.92		
Larimus sp.2	1.44		
Cynoscion phoxocephalus	3.77	Marine bottom-dwelling habitat	High
Isopisthus altipinnis	6.34	Benthopelagic habitat	High
Bairdiella sp. 1	0.24		
Sciaenidae sp.2	0.18		
Mentiicirrhus panamensis	0.27	Benthopelagic habitat	High
Stellifer sp.2	0.81		
Stellifer sp.1	0.42		
Larimus sp. 1	0.76		
Stellifer sp.4	0.07		
Stellifer zestocarus	0.45	Marine bottom-dwelling habitat	Low
Umbrina sp.	0.09		
Sciaenidae sp.1	0.37		
Cynoscion sp.	0.28		
Menticirrhus sp.3	0.04		
Macrodon mordax	0.05	Benthopelagic habitat	
Stellifer sp.3	1.38		
Cynoscion sp.1	0.41		
Larimus sp.4	0.03		
Menticirrhus sp.2	0.11		

Cynoscion sp.2	0.15		
Sciaenidae sp.3	0.01		
Sciaenidae sp.4	0.01		
**Gerreiformes**			
**Gerreidae**	2.32		
Eucinostomus sp.	0.87		
Gerriedae sp.	0.65		
Gerreidae sp.1	0.05		
Eucinostomus gracilis	0.75	Brackish bottom-dwelling habitat	Low
**Ephippiformes**			
**Ephippidae**	0.05		
Chaetodipterus zonatus	0.05		
**Lutjaniformes**			
**Haemulidae**	3.37		
Haemulidae sp.1	2.59		
Haemulon sp.	0.43		
Haemulidae sp.3	0.14		
Haemulidae sp.2	0.07		
Anisotremus sp.	0.07		
Hoplopagrus guentheri	0.03		
Haemulon sp. 2	0.04		
Microlepidotus brevipinnis	0.01	Benthopelagic habitat	High
**Lutjanidae**	0.03		
Lutjanus sp.	0.03		
**Lobotiformes**			
**Lobotidae**	0.07		
Lobotes pacifica	0.07	Benthopelagic habitat	High
**Tetraodontiformes**			
**Tetraodontidae**	0.09		
Sphoeroides lobatus	0.05	Brackish bottom-dwelling habitat	Medium
Sphoeroides sp.	0.01		
Tetraodontidae sp.	0.03		
**Centrarchiformes**			
**Kyphosidae**	0.03		
Kyphosidae sp.1	0.03		
**Perciformes**			
**Serranidae**	0.47		
Serranidae sp.	0.08		
Paralabrax sp.	0.01		
Paralabrax sp.3	0.14		
Diplectrum sp.	0.08		
Serranus sp.2	0.03		
Diplectrum sp.1	0.05		
Hemanthias sp.1	0.05		
Serranidae sp.1	0.03		
**N/N**	7.54		

Table S3. Biological parameters recorded at sampling stations during each survey campaign. Variables include taxa richness (R), abundance of fish larvae (N, individuals), Shannon diversity index (H), Pielou's evenness index (J), chlorophyll-a concentration (Clo-a, mg·m^{-3}), and zooplankton biomass (B, mg·m^{-3}). Values are presented for each station across campaigns conducted between 2010 and 2020.

Campaing	Station	R	N	H	J	Clo-*a*	B
2010-03	1	5	500	0.9254	0.575	0.007	1.54
	3	3	94	0.9772	0.8895	0.002	1.49
	5	6	726	1.642	0.9163	0.001	1.83
	6	5	232	1.185	0.736	0.002	1.61
	7	8	203	1.821	0.8755	0.004	1.54
	8	2	10	0.6931	1	0.014	1.76
	10	3	26	0.9586	0.8725	0.001	1.39
	11	1	11	0	0	0.001	1.46
	12	1	95	0	0	0.002	1.38
2011-04	1-	8	712	0.5154	0.2479	0.011	1.42
	2	3	10	0.9503	0.865	0.024	1.32
	3	4	16	1.213	0.875	0.012	1.06
	4	4	142	0.8796	0.6345	0.011	1.38
	5	9	773	1.156	0.5261	0.001	1.98
	6	9	370	0.5935	0.2701	0.006	1.61
	7	7	310	1.23	0.6319	0.006	1.34
	8	10	729	0.6881	0.2989	0.002	1.52
	9	9	105	1.34	0.61	0.001	1.32
	10	9	99	1.117	0.5084	0.002	1.52
	11	6	102	1.132	0.6316	0.003	1.47
	12	3	47	0.6103	0.5555	0.003	1.33
2012-05	1	18	188	2.406	0.8323	3.378	2.08
	3	8	47	1.557	0.7487	7.093	1.57
	4	18	1121	1.668	0.577	6.040	1.77
	5	11	1915	0.6591	0.2749	5.585	1.86
	6	5	86	0.8268	0.5137	1.638	1.42
	7	9	204	1.392	0.6336	2.652	1.92
	8	12	125	2.137	0.8599	1.776	1.44
	9	7	102	1.287	0.6615	2.118	1.65
	10	29	1176	1.608	0.4776	2.229	1.63
	11	14	139	2.214	0.839	2.207	1.41
	12	8	115	1.541	0.7412	2.075	2.23
	13	10	96	2.079	0.9027	1.246	1.89
2014-05	1	12	772	2.289	0.9213	1.975	1.85
	2	9	698	1.524	0.6935	1.560	1.71
	3	6	236	1.677	0.936	0.475	2.07
	4	9	8812	1.376	0.6264	1.486	1.86
	7	11	2214	1.858	0.7747	3.376	1.93
	19	5	590	0.9442	0.5866	2.201	1.51
	10	7	964	1.593	0.8189	1.934	1.56
	11	8	424	1.65	0.7937	1.350	2.24
	12	5	306	1.353	0.8408	2.933	1.91
	13	7	722	1.809	0.9297	0.980	1.75
	14	2	34	0.6931	1	1.166	2.08
	15	7	621	1.187	0.6097	3.282	2.24
2015-03	1	2	451	0.5629	0.8122	5.453	1.66

	2	3	281	0.8002	0.7284	4.548	1.78
	3	5	116	1.393	0.8656	5.659	1.91
	4	7	297	1.489	0.7653	3.713	1.66
	5	9	432	2.138	0.9732	4.103	1.69
	6	9	1440	1.683	0.7659	3.584	1.62
	7	4	4092	1.034	0.7456	5.719	1.74
	8	4	302	1.152	0.8308	5.341	2.01
	10	18	1677	2.745	0.9498	3.528	1.89
	11	6	228	1.732	0.9667	3.071	1.25
	12	11	541	2.131	0.8889	3.098	1.46
	13	9	428	1.781	0.8104	2.794	1.42
	15	8	2871	1.868	0.8981	3.542	1.19
2017-10	1	13	2397	2.09	0.8149	6.495	3.89
	2	7	1134	0.5962	0.3064	7.952	1.84
	3	3	405	0.51	0.4642	7.594	1.29
	4	6	1178	1.131	0.6313	9.304	2.00
	5	9	12780	0.3879	0.1766	8.043	1.95
	6	15	45274	0.6598	0.2436	4.387	2.34
	7	15	1362	2.482	0.9165	1.032	2.81
	8	10	1458	2.066	0.8971	1.074	1.91
	9	10	4760	1.443	0.6268	2.333	2.14
	10	3	988	0.7436	0.6768	2.335	1.74
	11	5	748	1.271	0.7896	4.829	1.63
	12	19	3313	2.183	0.7416	5.895	5.83
	13	11	1244	1.671	0.6971	5.102	1.83
	14	8	798	1.649	0.7932	2.795	1.96
	15	8	657	1.658	0.7973	1.857	1.64
2019-03	1	8	1004	1.956	0.9408	7.602	1.50
	2	3	1166	0.5847	0.5322	10.960	1.54
	3	3	226	0.8467	0.7707	9.826	1.38
	4	10	3509	2.015	0.875	9.331	1.56
	5	4	165	1.332	0.961	9.399	1.60
	6	11	2838	1.744	0.7273	6.686	1.48
	7	3	869	1.077	0.9808	6.268	1.37
	8	10	3097	1.872	0.8131	6.867	1.44
	9	11	7939	2.121	0.8847	4.762	1.45
	10	15	1097	2.546	0.94	5.948	1.63
	11	4	191	1.275	0.9195	4.891	1.43
	12	17	10127	2.052	0.7241	6.960	1.49
	13	9	630	1.783	0.8115	5.569	1.62
	14	14	7075	2.248	0.8518	4.762	1.37
	15	11	3740	2.106	0.8785	5.599	1.56
2019-08	1	1	182	0	0	0.063	1.49
	2	7	973	1.798	0.924	0.450	1.53
	3	4	594	1.237	0.892	0.037	1.37
	4	11	5452	1.412	0.5887	0.017	1.55
	5	4	3089	1.096	0.7909	0.027	1.59
	6	11	1808	1.654	0.6896	0.063	1.47
	7	3	2359	0.5022	0.4571	0.033	1.36
	8	7	1370	1.426	0.7329	0.030	1.43
	9	7	977	1.617	0.8308	0.020	1.44
	10	7	1274	1.629	0.837	0.017	1.61
	11	3	156	1.002	0.9123	0.047	1.42

	12	11	5569	1.378	0.5749	0.027	1.47
	13	19	9573	1.973	0.6701	0.023	1.61
	14	8	679	1.769	0.8507	0.143	1.36
	15	6	1044	1.603	0.8945	0.020	1.54
2020-02	2	3	1459	0.4828	0.4395	6.739	1.68
	3	2	143	0.2712	0.3912	9.256	1.33
	4	3	464	0.86	0.7828	6.823	1.77
	5	2	53	0.2676	0.386	3.216	2.08
	6	7	776	1.807	0.9288	4.863	1.67
	7	7	443	1.684	0.8653	4.443	1.79
	8	8	6804	1.979	0.9518	4.446	1.53
	12	5	365	0.9925	0.6167	5.268	3.65
	13	2	79	0.4471	0.6451	3.612	1.72
	14	6	617	1.435	0.8007	2.694	1.66
	15	7	351	1.889	0.9708	3.560	1.59
